# Effectiveness of 3D-printed orthoses for traumatic and chronic hand conditions: A scoping review

**DOI:** 10.1371/journal.pone.0260271

**Published:** 2021-11-18

**Authors:** T. A. M. Oud, E. Lazzari, H. J. H. Gijsbers, M. Gobbo, F. Nollet, M. A. Brehm

**Affiliations:** 1 Amsterdam UMC, University of Amsterdam, Rehabilitation Medicine, Amsterdam Movement Sciences, Amsterdam, The Netherlands; 2 Laboratory of Clinical Integrative Physiology, University of Brescia, Brescia, Italy; 3 Department of Clinical and Experimental Sciences, University of Brescia, Brescia, Italy; Medical University of Graz, AUSTRIA

## Abstract

**Background:**

In the field of orthotics, the use of three-dimensional (3D) technology as an alternative to the conventional production process of orthoses is growing.

**Purpose:**

This scoping review aimed to systematically map and summarize studies assessing the effectiveness of 3D-printed orthoses for traumatic and chronic hand conditions, and to identify knowledge gaps.

**Methods:**

The Cochrane Library, PubMed, EMBASE, CINAHL, Web of Science, IEEE, and PEDro were searched for studies of any type of 3D-printed orthoses for traumatic and chronic hand conditions. Any outcome related to the effectiveness of 3D-printed orthoses was considered. Two reviewers selected eligible studies, charted data on study characteristics by impairment type, and critically appraised the studies, except for case reports/series.

**Results:**

Seventeen studies were included: four randomized controlled trials, four uncontrolled trials, four case series and five case reports. Only three studies had a sample size >20. Impairments described were forearm fractures (n = 5), spasticity (n = 5), muscle weakness (n = 4), joint contractures (n = 2) and pain (n = 1). Four poor to fair quality studies on forearm fractures supported the effectiveness of 3D-printed orthoses on hand function, functionality, and satisfaction. One good quality study on spasticity demonstrated the effectiveness of 3D-printed orthoses on hand function. One poor quality pain study reported limited positive effects on satisfaction. Studies on muscle weakness and joint contractures showed no benefits.

**Conclusion:**

Current literature addressing the effectiveness of 3D-printed orthoses for traumatic and chronic hand conditions consists primarily of small and poor methodological quality studies. There is a need for well-designed controlled trials including patient-related outcomes, production time and cost analyses.

## Introduction

Hand function is important for the performance of activities. However, falls, cuts, or crush injuries may cause traumatic hand conditions, whereas chronic hand conditions can occur due to neuro-musculoskeletal disorders or long-lasting complaints resulting from traumatic hand conditions. Both types of hand conditions (including the wrist and fingers) may lead to impairments such as fractures, joint deformity, contractures, muscle weakness, spasticity, and/or pain [1–4]. These impairments may limit in performing activities of daily living like eating, dressing and writing, as well as work- and leisure-related activities [3–6]. Accordingly, this can seriously impact on participation and quality of life [5, 7, 8].

Orthoses, including casts, are commonly used in the treatment of traumatic and chronic hand conditions [9–11]. An orthosis is a rigid or semi-rigid device used for the purpose of support, alignment, prevention or correction of joint deformities, or to improve function or restrict motion of a movable body part [12]. For many centuries, plaster casts and, more recently, fiberglass casts have been used in the treatment of traumatic hand conditions [13, 14]. These casts are low cost, strong, and easy to apply [15], and research in distal radius fractures and ligament injuries has shown positive outcomes on bone healing, joint stability, pain reduction, joint motion, and muscle strength [14, 16]. Unlike traumatic hand conditions, where the orthosis is worn for a limited period of time, persons with chronic hand conditions mostly wear the orthosis permanently. Therefore, chronic hand conditions are commonly treated with custom fabricated orthoses of sustainable materials such as resin, leather, silicone or polypropylene [17]. In people with arthritis and post stroke, it has been shown that these orthoses can reduce impairments like pain, muscle weakness and spasticity, and increase the ability to use the affected hand in daily activities [18, 19].

Despite the benefits of casts and custom fabricated orthoses, complications and discomfort have also been reported, including skin lesions, improper fit, sweating due to low breathability, heavy weight, bulkiness, and not being waterproof [11, 15, 19]. Since casts and custom fabricated orthoses are handmade, the risks of complications and discomfort, especially skin lesions and improper fit largely depend on the practitioner’s skills and experience [11, 20]. Furthermore, the manufacturing of custom fabricated orthoses is a labor intensive and time consuming process [21].

In the last decade, the use of three-dimensional (3D) technology emerged in the field of orthotics, being a promising alternative to conventional orthoses. This technology involves three-dimensional scanning, modelling and printing, whereby materials are joined, layer by layer to manufacture 3D-printed orthoses [20]. So far, research into 3D-printed orthoses has mainly focused on the lower extremities, including two reviews on 3D-printed (ankle-)foot orthoses [21, 22]. These reviews concluded that 3D printing to manufacture (ankle-)foot orthoses seems to have potential benefits over conventional methods, in terms of improved comfort, fit and function. Furthermore, this technology allows to eliminate several steps from the conventional manufacturing process of custom fabricated orthoses, and may improve efficiency by a shorter production time and lower costs [20, 21, 23]. While previous studies on the effects of 3D-printed orthoses for the upper extremities also indicated some of these benefits [24–26], a synthesis of the results on the effectiveness of 3D-printed orthoses for the upper extremities, specifically traumatic and chronic hand conditions is currently lacking.

A preliminary literature search conducted on September 4 2020, in PubMed, JBI Evidence Synthesis, Open Science Framework, the Cochrane Database of Systematic Reviews and the PROSPERO database identified that to date, no scoping or systematic reviews on 3D-printed hand orthoses have been performed and none are currently underway. Since the use of 3D printing in manufacturing hand orthoses is quite recent and literature lacks high quality and homogeneous studies to perform a systematic review, we decided to perform a scoping review. The objective was to systematically map and summarize the research done on the effectiveness of 3D-printed orthoses for traumatic and chronic hand conditions, and identify any existing gaps in knowledge and needs for future research.

## Methods

This review was conducted in accordance with the JBI methodology guidance for scoping reviews, using the Preferred Reporting Items for Systematic Reviews and Meta-Analyses–Scoping Reviews (PRISMA-ScR) checklist [27]. The protocol was registered on September 4 2020, with the Open Science Framework (https://osf.io/t9rxn/).

### Eligibility criteria

#### Population

We included studies on participants of any age with traumatic or chronic hand (including wrist and fingers) conditions, respectively due to traumatic injuries or chronic neurological, neuromuscular or musculoskeletal disorders.

#### Interventions

We focused on all types of 3D-printed hand orthoses, whether as a single intervention or combined with other interventions. Studies using orthoses with only small 3D-printed parts, and studies on 3D-printed prostheses and myoelectric orthoses were excluded. In order to be fully inclusive, studies involving any type of comparator or even none were included.

#### Outcome measures

We included each outcome measure related to the effectiveness of 3D-printed hand orthoses, and also inventoried reported adverse events.

#### Types of studies

Primary research articles of all types of study designs were included. Studies were restricted to the English language, and only full-text publications were included. Ongoing studies, conference abstracts and posters were excluded.

### Search strategy

A preliminary limited search of The Cochrane Library and PubMed databases was conducted by two reviewers (EL, TO) to identify appropriate keywords and medical subject headings (MeSH). Subsequently, we formulated a broad search strategy for PubMed combining the keywords and MeSH terms related to 1) 3D-printing, 2) upper extremity body parts and 3) orthoses (S1 Appendix). This search strategy was adapted for the other indexed databases. On September 17 2020, a literature search was conducted by one reviewer (EL) on the following databases: The Cochrane Library, PubMed, EMBASE, Web of Science, IEEE, CINAHL and PEDro. This search was updated on January 30 2021.

The retrieved search results were listed in Rayyan, a web-based literature screening program [28], and duplicates were removed. The search was supplemented through scanning the reference lists of included studies.

### Selection of studies

Two reviewers (EL, TO) independently screened titles and abstracts using the predetermined eligibility criteria to include or exclude studies. Each excluded article was labeled with an exclusion reason in Rayyan. If there was any doubt, the full-text was retrieved. To resolve uncertainties about potentially relevant studies, the reviewers directly contacted the authors. Conflicts regarding inclusion status were resolved by discussion, but if no consensus was achieved, a third reviewer (MB) made the final decision. A PRISMA flow diagram was used to give an overview of the study selection process.

### Data extraction

Each study was charted by one reviewer (EL) using a data extraction table designed in Microsoft Excel. The charted data was verified by a second reviewer (TO), after which the data extraction table was refined. The following characteristics were extracted: study type, subjects (number, age, diagnosis), intervention(s) (orthosis type, duration of wearing), comparator, and measurement time points.

### Critical appraisal of studies

To interpret the results along with the knowledge about the methodological quality of the included studies, the randomized controlled trials (RCTs) and non-randomized studies (NRSs) except for case series/reports were critically appraised. We used the Modified Downs and Black checklist for the critical appraisal as it can be applied to assess the methodological quality of both RCTs and NRSs [29]. The checklist contains 27 items grouped in five sections; reporting, external validity, internal validity-bias, internal validity-confounding, and power. Two reviewers (EL, TO) independently assessed the studies. Disagreements were resolved with a consensus procedure, if necessary, with the third reviewer (MB). The maximum score achievable for RCTs is 28 and for NRSs it is 24, since items 21–24 are not applicable. To guide interpretation of results, scores ≥24 were considered as excellent quality; scores 20–23 good quality; scores 15–19 fair quality; scores ≤14 poor quality [30].

### Synthesis of results

For traumatic and chronic hand conditions separately, we grouped studies by type of impairment. Data were narratively synthesized by reporting the number of studies for each impairment type, sample size, associated diagnoses, and type of orthoses provided. Key findings were presented by assessed outcomes. Identified research gaps in the existing literature were addressed in the Discussion.

## Results

**Fig 1:**
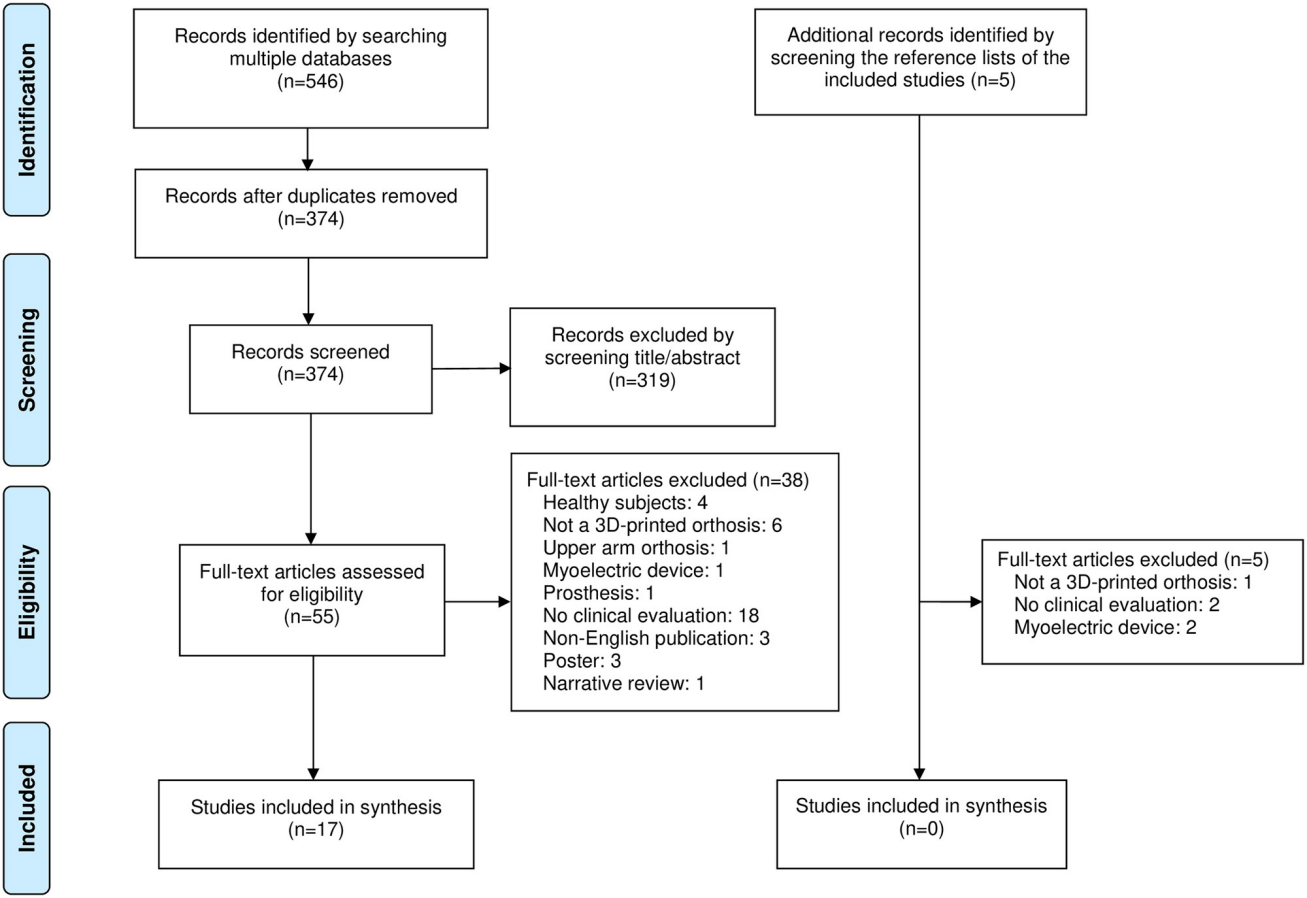
PRISMA-ScR flow diagram.

The selection process of the search results is presented in a PRISMA-ScR flow diagram (Fig 1). The searches of the electronic databases yielded 546 records. After duplicates were removed, the titles and abstracts of 374 records were screened. Subsequently, 55 full-text articles were retrieved and assessed for eligibility. Seventeen studies (published between 2017 and 2020) fulfilled the eligibility criteria [24–26, 31–44]. After checking the reference lists of these studies, five articles were considered potentially relevant, but none of them fulfilled the eligibility criteria.

### Characteristics of included studies

Of the seventeen included studies, four were RCTs [25, 26, 33, 40], and thirteen were NRSs, including four uncontrolled clinical trials (UCTs) [24, 32, 34, 38], four case series [41–44], and five case reports [31, 35–37, 39] (Table 1). Sample sizes ranged from 1 to 60 participants. Only three studies had a sample size >20 [25, 26, 33]. For traumatic hand conditions, only studies on forearm fractures were identified (n = 5) [24, 31–34], whereas studies on chronic hand conditions (n = 12) targeted spasticity [26, 35–38], muscle weakness [26, 39–42], joint contractures [43, 44], and pain [25]. Of the four types of 3D-printed orthoses reported, wrist-hand orthoses (WHOs) were the most frequently investigated. Ten studies (59%) did not use a comparator. Characteristics of each study and information regarding the 3D printing process, tabulated by impairment type, are presented in Table 2. Notable are the many variations in design within the four orthoses types.

**Table 1:** Study characteristics overview.

	n/N	(%)
Type of study		
*Randomized controlled trial*	4/17	(24%)
*Non-randomized study*	13/17	(76%)
*Uncontrolled clinical trial*	4/13	(31%)
*Case series*	4/13	(31%)
*Case report*	5/13	(38%)
Sample size >20	3/17	(18%)
Traumatic hand conditions	5/17	(29%)
*Forearm fractures*	5/5	(100%)
Chronic hand conditions	12/17	(71%)
*Spasticity*	5/12	(42%)
*Muscle weakness*	4/12	(33%)
*Contractures*	2/12	(17%)
*Pain*	1/12	(8%)
Type of orthosis		
*Wrist-hand-finger orthosis*	4/17	(24%)
*Wrist-hand orthosis*	9/17	(53%)
*Hand-finger orthosis*	2/17	(12%)
*Finger orthosis*	2/17	(12%)
Comparator type		
*No comparator*	10/17	(59%)
*Non-use of orthosis*	3/17	(18%)
*Prefabricated orthosis*	2/17	(12%)
*Low-temperature orthosis*	1/17	(6%)
*Cast and conventional orthosis*	1/17	(6%)

**Table 2:** Study characteristics by type of impairment.

Author, Year	Study Design	Subjects N included (n analyzed), age, diagnosis	3D-printing process	Intervention (I) and Comparator (C) orthosis type and wearing time	Co-intervention	Baseline and follow-up
**Traumatic hand conditions**						
***Forearm fractures***						
Abreu de Souza et al. 2017 [31]	Case Report	n = 1 (1), 24 yrs, distal radius fracture	Geometry acquisition: hand-held 3D laser scanner and MeshLab software Design: freely available online models 3D printing: not specified Material: PLA	I: Static circular 3D-printed WHO, 45 days C: No comparator	Surgery prior to 3D-printed orthosis prescription	One-time point measurement
Chen et al. 2017 [32]	UCT	n = 10 (10), range 5–78 yrs, distal forearm fracture	Geometry acquisition: CT or MRI of both arms Design: self-designed software 3D printing: SLS or stereo lithography Material: PP and PA	I: Static circular 3D-printed WHO, 6 weeks C: No comparator	1 week plaster cast prior to 3D-printed orthosis prescription	T0 = 2 weeks T1 = 6 weeks T2 = 7 weeks
Chen et al. 2020 [33]	RCT	n = 60 (60), range 5–78 yrs, distal forearm fracture	Geometry acquisition: CT or MRI of both arms Design: Self-designed software, Solidworks 2015, Workbench 18.0 3D printing: SLS Material: PA	I: Static circular 3D-printed WHO, 5 weeks C: Group 1: plaster cast, 6 weeks Group 2: conventional orthosis, 6 weeks	1 week plaster cast prior to 3D-printed orthosis prescription	T0 = 2 weeks T1 = 6 weeks T2 = 3 months
Guida et al. 2019 [24]	UCT	n = 18 (18), mean age 11.9 yrs, nondisplaced metaphyseal distal radius fracture	Geometry acquisition: 3D laser scanner Design: Rhinoceros v5 software 3D printing: FDM Material: thermoplastic modified ABS and polycarbonate	I: Static circular 3D-printed WHO, 4 weeks C: No comparator	48-72h immobilization prior to 3D-printed orthosis prescription	T0 = Baseline T1 = 4 weeks
Janzing et al. 2020 [34]	UCT	n = 5 (3), age ≥ 50 yrs, dorsally dislocated distal radius fracture	Mirrored geometry acquisition: 3D optical scanner Design: Blender open source software 3D printing: FDM Material: PLA	I: Static 3-point 3D-printed WHO, 5 weeks C: No comparator	None	T0 = 2–3 days T1 = 1 week T2 = 3 weeks T3 = 5 weeks
**Chronic hand conditions**						
***Spasticity***						
Lee et al. 2018 [35]	Case Report	n = 1 (1), 19 yrs, hemiparesis and spasticity post subdural hematoma	Mirrored geometry acquisition: 3D optical scanner Design: Geomagic Freeform Software 3D printing: FFF Material: TPU	I: Static 3D-printed WHO with 3D-printed assistive devices (pen holder, typing device), 1 month C: Prefabricated assistive orthosis	None	One-time point measurement
Rosenmann et al. 2017 [36]	Case Report	n = 1 (1), child, unknown age, upper limb spasticity due to cerebral palsy	Geometry acquisition: 3D scanned plaster cast Design: 3ds MAX software 3D printing: not specified Material: PLA	I: Static volar 3D-printed WHFO, wearing time not reported C: No comparator	None	One-time point measurement
Schmitz et al. 2019 [37]	Case Report	n = 1 (1), 11 yrs, hand spasticity due to cerebral palsy	Geometry acquisition: plaster cast scanned with 3D hand-held laser scanner Design: Meshmixer software 3D printing: FDM Material: PETG	I: Static circular 3D-printed WHO, wearing time not reported C: Non-use of orthosis	None	One-time point measurement
Wang et al. 2018 [38]	UCT	n = 18 (13), mean age 68.3±4.9 yrs, hand spasticity post stroke	Mirrored geometry acquisition: hand palm sand mold scanned with hand-held 3D optical scanner Design: 3D Max software 3D printing: FDM Material: PLA	I: Static volar 3D-printed HFO after daily rehabilitation training, 3 months 3x ±2h/day C: No comparator	Rehabilitation training	T0 = Baseline T1 = 3 weeks T2 = 3 months
Zheng et al. 2020 [26]	RCT	n = 44 (40), adults, wrist flexor spasticity post stroke	Geometry acquisition: optical scanner Design: Unigraphics NX 8.0 software 3D printing: not specified Material: light-activated resin	I: Static circular 3D-printed WHFO, 6 weeks 4–8 h/day C: Volar low-temp thermoplastic WHFO, 6 weeks 4–8 h/day	Conventional rehabilitation, 40 min 5x/week for 6 weeks	T0 = Baseline T1 = 3 weeks T2 = 6 weeks
***Muscle weakness***						
Chae et al. 2020 [42]	Case Series	n = 2 (2), 55, 59 yrs, neuropathy 1. carpal tunnel syndrome 2. ulnar neuropathy wrist after surgery	Geometry acquisition: CT + MIMICS Medical v17 software Design: Geomagic Freeform Software 3D printing: FFF Material: TPU	I: 1. Static radial 3D-printed WHO, 2 weeks 2. Static semi-circular 3D-printed WHO, 8 weeks C: No comparator	1. None 2. Surgery prior to 3D-printed orthosis prescription	T0 = Baseline T1 = 1. 2 weeks 2. 8 weeks
Chang et al. 2018 [39]	Case Report	n = 1 (1), 33 yrs, upper extremity motor impairment post stroke	Mirrored geometry acquisition: hand-held 3D scanner Design: Computer Aided Design software 3D printing: FDM Material: PLA	I: Dynamic dorsal 3D-printed WHFO, 1 month during functional training C: No comparator	None	T0 = Baseline T1 = 1 month
Huang et al. 2019 [40]	RCT	n = 10 (10), age >20 yrs, upper limb hemiparalysis post stroke	Mirrored geometry acquisition: 3D scanner Design: Meshmixer software 3D printing: not specified Material: not specified	I: Task-oriented approach (TOA) for upper limb training wearing dynamic dorsal 3D-printed HFO, 30 min 2x/week for 4 weeks Thereafter, 2-week home program (≥30 min/day) C: Same TOA and home program as intervention group, non-use of orthosis	None	T0 = Baseline T1 = 4 weeks T2 = 6 weeks
Portnova et al. 2018 [41]	Case Series	n = 3 (at T1 n = 2), limited mobility digits, able to extend wrist against gravity due to spinal cord injury	Geometry acquisition: tape measure Design: SolidWorks software 3D printing: FFF Material: PLA	I: Dynamic wrist driven dorsal 3D-printed WHFO, 10 min C: Non-use of orthosis	None	T0 = 2nd visit T1 = 3rd visit
***Joint contractures***						
Arulmozhi et al. 2018 [44]	Case Series	n = 3 (3), 46, 55, 63 yrs, rheumatoid arthritis 1. and 2. Boutonniere deformed and swollen digits 3. swan neck deformity	Geometry acquisition: vernier caliper Design: Solidworks 2013 software 3D printing: FDM Material: ABS or Flex-PLA	I: 1. Static circular 3D-printed FO 2. and 3. Static 3-point 3D-printed FO Wearing time not reported C: No comparator	None	T0 = 1 week T1 = 1 month
Nam et al. 2018 [43]	Case Series	n = 3 (3), 21, 39, 37 yrs, post burn 1. deformity all digits. 2. claw hand deformity digits 3–5, 3. mallet finger deformity 2^nd^ digit	Geometry acquisition: ruler Design: Thingiverse, and Rhinoceros 5.0 or Simplify3D software 3D printing: FDM Material: PLA or TPU	I: 1. Static 3D-printed FO digit 2 and 5, 24h/d 2. Static 3-point 3D-printed FO digit 3 and 4 3. Static 3-point 3D-printed FO Wearing time not reported for cases 2 and 3 C: No comparator	1. Other rehabilitation management 3. Prior to 3D-printed FO, plastic orthosis which gave skin irritation	T0 = Baseline T1 = 18 months
***Pain***						
Kim et al. 2018 [25]	RCT	n = 22 (20), adults, overuse syndrome in upper wrist area	Geometry acquisition: held-hand 3D scanner Design: Geomagic Touch and Freeform software 3D printing: FFF Material: TPU	I: Static circular 3D-printed WHO, 1 week C: Prefabricated WHO, 1 week	None	T0 = Baseline T1 = 1 week

Abbreviations: RCT: randomized controlled trial, UCT: uncontrolled clinical trial, WHO: wrist-hand orthosis, WHFO: wrist-hand-finger orthosis, HFO: hand-finger orthosis, FO: finger orthosis, CT: Computed Tomography, MRI: Magnetic Resonance Imaging, SLS: selective laser sintering, FDM: fused deposition modeling, FFF: fused filament fabrication, ABS: acrylonitrile butadiene styrene, PETG: polyethylene terephthalate glycol, PLA: polylactic acid, TPU: thermoplastic polyurethane, PP: polypropylene, PA: polyamide.

### Results of critical appraisal

Four RCTs [25, 26, 33, 40] and four UCTs [24, 32, 34,38] were critically appraised. The quality scores, presented in Table 3, ranged from 11 to 21. With a score of 21/28, the RCT of Zheng et al. was considered of good methodological quality [26]. This was the only study that reported a power calculation, although it was found to be insufficient. Most RCTs and UCTs did not consider any confounders. Scores were low for blinding and the overall external validity, and concealment of allocation treatment was unclear in three of four RCTs [25, 33, 40]. Additionally, three of four UCTs did not undertake statistical analyses [32, 34, 38].

**Table 3:** Critical appraisal of studies.

Study	Reporting										External validity			Internal validity–bias							Internal validity–confounding						Power	Quality score
	1	2	3	4	5*	6	7	8	9	10	11	12	13	14	15	16	17	18	19	20	21	22	23	24	25	26	27	
**RCT**																												
Chen et al. [33]	1	1	1	1	1	1	1	1	1	1	0^u^	0^u^	0^u^	0	0	1	1	1	1	0	0^u^	1	1	0^u^	0	1	0^u^	**17 Fair**
Huang et al. [40]	1	1	1	1	0	1	0	0	1	1	0^u^	0^u^	0^u^	0	0^u^	1	1	1	1	1	0^u^	0^u^	1	0^u^	0	1	0^u^	**14 Poor**
Kim et al. [25]	1	1	1	1	0	1	1	0	1	1	0^u^	0^u^	0^u^	0	0^u^	1	1	1	0^u^	1	0^u^	0^u^	1	0^u^	0	1	0^u^	**14 Poor**
Zheng et al. [26]	1	1	1	1	0	1	1	1	1	1	0^u^	0^u^	1	0	1	1	1	1	1	1	1	1	1	1	0	1	0	**21 Good**
**UCT**																												
Chen et al. [32]	1	1	1	1	1	0	0	1	1	0	0^u^	0^u^	0^u^	0	0	1	1	0	1	0	NA	NA	NA	NA	0	1	0^u^	**11 Poor**
Guida et al. [24]	1	1	1	1	0	1	1	1	1	1	0^u^	0^u^	0	0	0	1	1	1	1	1	NA	NA	NA	NA	0	1	0^u^	**15 Fair**
Janzing et al. [34]	1	1	1	1	0	1	0	1	1	0	0^u^	0^u^	0^u^	0	0	1	1	0	1	1	NA	NA	NA	NA	0	1	0^u^	**12 Poor**
Wang et al. [38]	0	1	1	1	0	1	1	1	1	0	0^u^	0^u^	1	0	0^u^	1	1	0^u^	0	1	NA	NA	NA	NA	0	0	0^u^	**11 Poor**

RCT: randomized controlled trial, UCT: uncontrolled clinical trial.

Item scores: 1 = Yes; 0 = No; 0^u^ = Unable to determine; NA = Not applicable.

*item 5: 2 = Yes; 1 = Partially; 0 = No.

### Synthesis of results

Identified outcomes related to the effectiveness of 3D-printed orthoses were hand function, functionality, satisfaction, production time, and costs. Furthermore, adverse events were reported. Hand function included the sub-items pain, range of motion (ROM), pinch and grasp force, motor function, and spasticity. Functionality included the sub-items manual dexterity, performance in activities of daily living (ADL), and disability in ADL. An overview of the outcomes as assessed in each study is presented in Table 4.

**Table 4:** Outcomes investigated in the included studies.

Study	Hand function							Functionality			Participants’ satisfaction	PT	PC	Adverse events
	Pain	ROM	Pinch force	Grasp force	Motor function	Spasticity	Swelling	Manual dexterity	Performance in ADL	Disability in ADL				
**Forearm fractures**														
Abreu de Souza et al. [31]												**✓**	**✓**	
Chen et al. [32]											**✓**			**✓**
Chen et al. [33]	**✓**	**✓**		**✓**							**✓**			**✓**
Guida et al. [24]	**✓**									**✓**	**✓**			
Janzing et al. [34]	**✓**									**✓**	**✓**			**✓**
**Spasticity**														
Lee et al. [35]									**✓**		**✓**			
Rosenmann et al. [36]											**✓**		**✓**	
Schmitz et al. [37]									**✓**			**✓**		
Wang et al. [38]	**✓**	**✓**		**✓**	**✓**	**✓**								
Zheng et al. [26]	**✓**	**✓**			**✓**	**✓**	**✓**				**✓**			
**Muscle weakness**														
Chae et al. [42] *			**✓**	**✓**					**✓**		**✓**			
Chang et al. [39]					**✓**									
Huang et al. [40]			**✓**	**✓**				**✓**						
Portnova et al. [41]			**✓**					**✓**	**✓**		**✓**	**✓**	**✓**	
**Joint contractures**														
Arulmozhi et al. [44]		**✓**									**✓**			
Nam et al. [43]		**✓**								**✓**				
**Pain**														
Kim et al. [25]	**✓**								**✓**		**✓**			

ROM: range of motion, ADL: activities of daily living, PT: production time, PC: production costs.

* Chae et al. reported a VAS score, however it was not specified which item was scored. Despite that authors were contacted, this information could not be obtained.

#### Traumatic hand conditions

*Orthoses for forearm fractures*. Of the five studies targeting forearm fractures, four examined the effects of a 3D-printed circular WHO [24, 31–33], and one of a 3-point WHO [34].

*Hand function*. Hand function was reported in three of five studies. In Chen’s RCT, pain, ROM, grasp force and return to activity were collectively assessed with the Cooney modification of the Green and O’Brien score [33]. The 3D-printed orthosis group scored significantly better (85% had good/excellent results) compared to the plaster cast group (65%, p = 0.014) and the conventional orthosis group (70%, p = 0.035). Guida’s UCT assessed pain with the pain subscale of the Patient-Rated Wrist Evaluation (PRWE) and Visual Analogue Scale (VAS), reporting a significant decrease of pain after four weeks of treatment with the 3D-printed circular WHO (PRWE-pain: mean difference (MD) 19.7; VAS: MD 5.48, p<0.001) [24]. In Janzing’s UCT, two of three participants had complete pain relief on the 100mm VAS after five weeks of immobilization with the 3D-printed 3-point WHO, while the third participant reported pain increase caused by a pressure point [34].

*Functionality*. Disability in ADL was assessed in two studies [24, 34]. Guida’s study reported a significant improvement on the PRWE function subscale after treatment with the 3D-printed circular WHO (MD 17.7, p<0.001) [24]. Janzing et al. used the Katz-index, showing that after three weeks of immobilization with the 3D-printed 3-point WHO, all three participants were independent in ADL [34].

*Satisfaction*. Four studies assessed satisfaction [24, 32–34]. Chen’s UCT used a self-designed questionnaire, scoring 11.5 (15 = highest score) with wearing a 3D-printed circular WHO [32]. In their RCT, using the same questionnaire, satisfaction scored significantly higher for the 3D-printed orthosis group (8.65±1.040) compared to the plaster cast (6.85±1.137) and conventional orthosis group (8.10±1.252) (p≤0.001) [33]. Guida et al. reported good satisfaction with wearing a 3D-printed WHO for two items assessed (both scored 4/5 points) [24]. Janzing’s UCT used a 100mm VAS, and reported positive scores on wearing comfort of the 3-point WHO for two participants (90/100mm) and a negative score for one participant (10/100mm) because of a pressure point [34].

*Production time and costs*. One case report from Brazil showed that printing time of the circular 3D-printed WHO was 45 minutes and material costs were 2.40 USD [31].

*Adverse events*. Of all the included studies, only three measured adverse events [32–34]. Chen’s UCT investigated pressure sores, stability of immobilization, blood circulation and pressure-related discomfort of the 3D-printed WHO with a questionnaire. The mean score was 9.8 (12 = no complications) [32]. In Chen’s RCT, the 3D-printed group had significantly less complications compared to the plaster cast and conventional orthosis groups (p = 0.005), although the scores of separate items did not differ between groups [33]. Janzing et al. excluded two of five participants because of a secondary fracture displacement. Another participant reported a pressure point with skin redness and pain [34].

#### Chronic hand conditions

*Orthoses for spasticity*. Five studies evaluated 3D-printed orthoses for wrist and/or hand flexor spasticity, caused by stroke [26, 38], cerebral palsy [36, 37], and a subdural hematoma [35]. In these studies, the effects of wrist-hand-finger orthoses (WHFOs), WHOs, and hand-finger orthoses (HFOs) were examined.

*Hand function*. Two studies assessed hand function in terms of spasticity, pain, ROM and motor function [26, 38]. Zheng’s RCT showed a significant reduction of spasticity on the Modified Ashworth Scale in stroke patients receiving a 3D-printed WHFO and conventional rehabilitation compared to those receiving a thermoplastic WHFO and conventional rehabilitation after six weeks (Z = -0.681, p = 0.02) [26]. In Wang’s UCT, also in stroke survivors, a significant reduction of spasticity between baseline and three months of wearing a 3D-printed HFO (p<0.05) was found [38].

Regarding pain, Zheng et al. found no difference on the VAS after six weeks treatment between 3D-printed WHFOs and thermoplastic WHFOs (p = 0.637) [26]. Wang et al. also found no difference on the VAS after three months of wearing a 3D-printed HFO [38].

For ROM, Zheng’s study showed significantly improved passive wrist extension (MD 7.0 degrees, p<0.001) and ulnar deviation (MD 4.2 degrees, p = 0.028) for 3D-printed WHFOs compared to thermoplastic WHFOs, while wrist flexion (p = 0.194) and radial deviation (p = 0.303) did not differ [26]. Wang’s UCT reported no difference in active and passive ROM of the wrist and fingers after using a 3D-printed HFO. Also, grasp force showed no difference [38].

Motor function, assessed in Zheng’s RCT with the wrist and hand subscales of the Fugl-Meyer Assessment-Upper Extremity (FMA-UE), significantly improved in the group wearing 3D-printed WHFOs compared to those wearing thermoplastic WHFOs (MD 1.3, p<0.001) [26].

Wang et al. used the Brunnstrom approach, showing a significant improved hand movement pattern (p<0.05) with wearing 3D-printed HFOs [38].

In addition, Zheng et al. measured swelling with a four-point scale, reporting a significant decrease in favour of the group wearing 3D-printed WHFOs (Z = -4.806, p<0.001) [26].

*Functionality*. In Lee’s case report, performance in ADL of a patient with a subdural hematoma was evaluated for three tasks of the Jebsen Hand Function Test (JHFT) after using a 3D-printed WHO for one month, showing a clear reduction in time needed on the simulated feeding task [35]. In Schmitz’s case report on a patient with cerebral palsy, improvements in 3/7 JHFT subtests were observed, and a total reduction of 58 seconds while wearing the 3D-printed WHO compared to no orthosis [37].

*Satisfaction*. Three studies reported on satisfaction. Zheng’s RCT used the Quebec User Evaluation of Satisfaction with Assistive Technology (QUEST) questionnaire, showing no significant difference between stroke patients using 3D-printed WHFOs and those using thermoplastic WHFOs (p = 0.243) [26]. The device component of the QUEST was used in Lee’s case report, showing higher scores for satisfaction on all three assessed JHFT tasks for the 3D-printed WHO compared to the prefabricated orthosis [35]. In Rosenmann’s case report on a child with cerebral palsy, the 3D-printed WHFO was described as fun to use, fashionable, light and customizable, yet it was hard to wear and remove, smelly and lead to pressure points [36].

*Production time and costs*. Schmitz’s case report indicated that the entire production process of the 3D-printed WHO took 23 hours [37]. Rosenmann et al. reported an estimated cost of 10 USD for their 3D-printed WHFO [36].

*Orthoses for muscle weakness*. Four studies evaluated 3D-printed orthoses for wrist and/or hand muscle weakness, caused by stroke [39, 40], spinal cord injury [41], and peripheral nerve injuries [42]. Types of orthoses evaluated were dynamic dorsal 3D-printed WHFOs and HFOs, and a static 3D-printed WHO.

*Hand function*. Three of four studies evaluated muscle force [40–42]. In Huang’s RCT in stroke survivors, palmar pinch force at six weeks significantly increased in the group wearing a 3D-printed HFO in addition to a task-oriented approach and homework program compared to baseline (p = 0.041), while no significant change was noted in the group only receiving a task-oriented approach and homework program and between groups. Lateral pinch force and grasp force significantly increased in both groups, but did not differ between groups [40]. In Portnova’s case series in spinal cord injury, two participants increased their pinch force by 122.2% and 13.3% while wearing a 3D-printed WHFO compared to no orthosis, and the third participant was able to perform this grasp for the first time [41]. In Chae’s case series, both participants with peripheral nerve injury improved 6 kilos in grasp force after using the WHO, and one of them also improved 2 and 1 kilos in respectively lateral and pinch force [42]. Chang et al. used the FMA-UE to assess motor function in a stroke survivor, reporting an improvement in score from 15 to 19 after using the 3D-printed WHFO [39].

*Functionality*. Two studies examined manual dexterity, assessed with the Box and Blocks Test (BBT) [40, 41]. Huang’s study observed no significant difference for stroke survivors wearing 3D-printed WHOs in addition to a task-oriented approach and homework program compared to the group wearing no orthosis [40]. In the case series in spinal cord injury, two of three users improved on the BBT while wearing the 3D-printed WHFO compared to no orthosis [41]. Two participants also improved on performance in ADL as assessed with the JHFT. Chae et al. used showed a decrease in JHFT total time for both participants with peripheral nerve injury after wearing the 3D-printed WHO [42].

*Satisfaction*. Two case series assessed satisfaction [41, 42]. In the study on spinal cord injury, patients rated the 3D-printed WHFO in terms of function, aesthetics and comfort on a 10-point scale, resulting in average scores of 6.8, 7.7, and 7.7 [41]. Chae et al. used the Korean QUEST 2.0, showing a score of 4.62 and 4.08 out of 5 for the 3D-printed WHO in both peripheral injury patients [42].

*Production time and costs*. The case series on spinal cord injury from the United States reported that production time of the 3D-printed WHFO took 8–9.2 hours and cost were 15–20 USD for materials, while production time of the conventional metallic orthosis took 11 hours and cost were 140 USD [41].

*Orthoses for joint contractures*. Two case series examined the effect of 3D-printed finger orthoses (FOs) for finger joint contractures due to burn injury [43] and rheumatoid arthritis [44].

*Hand function*. Both case series assessed hand function in terms of ROM. The study in rheumatoid arthritis found no difference in finger joint ROM with wearing the 3D-printed FO [44]. Nam et al. reported improvements of active finger flexion and extension in two of three hand burn patients with wearing the 3D-printed FO [43].

*Functionality*. In the case series in hand burn patients, disability in ADL was measured with the Modified Barthel Index in two of the three participants. The total score improved for the first user (84 to 91/100), but not for the second user (95/100) [43].

*Satisfaction*. The case series in rheumatoid arthritis assessed satisfaction, reporting that one user felt that the FO had a correct fit, reduced stiffness, but was heavy weight. The second user considered the FO comfortable and lightweight and easy to use. The third user experienced an initial malaise, but felt comfortable gradually [44].

*Orthoses for pain*. One RCT focused on the effects of 3D-printed WHOs on wrist pain in overuse syndrome [25].

*Hand function*. Wrist pain was assessed with the PRWE pain subscale, showing no significant difference between 3D-printed WHOs compared to prefabricated WHOs.

*Functionality*. Performance in ADL was assessed with the JHFT. No difference was noted in overall score. The 3D-printed WHO group was significantly slower on the simulated feeding task (p = 0.01).

*Satisfaction*. Satisfaction was assessed with the Orthotics and Prosthetics Users’ Survey (OPUS). The 3D-printed WHO group showed significant improvements compared to the prefabricated WHO group on 2/28 items; “Put toothpaste on brush and brush teeth” (p = 0.036) and “Dial a touch tone phone” (p = 0.004).

## Discussion

This scoping review summarized the literature investigating the effectiveness of 3D-printed orthoses for traumatic and chronic hand conditions, identifying 17 studies meeting the inclusion criteria. The vast amount of studies (n = 12) focused on 3D-printed orthoses for different types of impairments in chronic hand conditions.

### Amount and quality of evidence

The current body of evidence is represented by a small number of studies, indicating a limited amount of research on 3D-printing to manufacture hand orthoses. Apparently, there is a growing interest in manufacturing 3D-printed hand orthoses, as the included studies were all published in the last four years. This novel technique is in an exploratory phase, as illustrated by the large proportion of case series and case reports (53%), mainly on 3D-printed orthoses for chronic hand conditions. Yet, these study types have a low level of evidence. The quality of evidence of the 4 RCTs [25, 26, 33, 40] and 4 UCTs [24, 32, 34, 38] was rated fair or poor in 7/8 studies, which likely influences the reliability of the results. Of all studies, mostly of small sample sizes, only one RCT showed good methodological quality [26].

Considering the methodological quality of the outcome measures studied, hand function and functionality were assessed with validated tools. Pain, measured with the VAS and PRWE, was the most frequently assessed hand function impairment. Functionality, the least often reported outcome, was mostly evaluated with the JHFT. Regarding satisfaction, three studies used the QUEST [26, 35, 42] and one study the satisfaction module of the OPUS [25], which are both validated and reliable tools [45]. Seven studies used a self-designed method to assess satisfaction, which may have influenced the reliability of the obtained results [24, 32–34, 36, 41, 44]. Only three case reports [31, 36, 37] and one case series [41] reported production time and costs, so information on these topics was limited. However, for the implementation of state-of-the-art technology like 3D-printed orthoses, information on cost- and time-savings besides the effectiveness is important [46] and should be assessed in future studies.

### Summary of main results

The case reports and case series included in this review evaluated different types of 3D-printed hand orthoses, used non-validated tools to assess the outcomes and are of low level of evidence. Consequently, only the main findings of the clinical trials were summarized and discussed.

#### Traumatic hand conditions

*Orthoses for forearm fractures*. Guida’s UCT and Chen’s RCT reported significant improvements on hand function [24, 33]. Since a composite score was used in Chen’s RCT of fair methodological quality, it cannot be determined though which item(s) improved [33]. The fair and poor methodological quality UCTs of Guida et al. and Janzing et al. demonstrated a positive effect on pain [24, 34]. However, bone healing generally occurs within four weeks of immobilization, reducing pain naturally. Since both studies were uncontrolled, the improvement cannot be merely attributed to the specific use of the 3D-printed orthosis. Both studies also reported positive findings on disability in ADL. Satisfaction was positively assessed in all four studies. Whether 3D-printed WHOs result in less adverse events than plaster casts and conventional orthoses is questionable, as the overall score in Chen’s RCT showed a significant difference in contrast to the scores of each separate item [33].

#### Chronic hand conditions

*Orthoses for spasticity*. The RCT and UCT on 3D-printed orthoses for spasticity could not be compared because of too much heterogeneity [26, 38]. Wang’s poor methodological quality UCT demonstrated a significant improved movement pattern and spasticity reduction after using a 3D-printed HFO [38]. Zheng’s good methodological quality RCT showed that 3D-printed WHFOs combined with rehabilitation therapy significantly gives better outcomes on spasticity, ROM, motor function and swelling than thermoplastic WHFOs combined with rehabilitation, while there was no benefit on satisfaction [26].

*Orthoses for muscle weakness*. Huang’s poor methodological quality RCT demonstrated that wearing a dynamic 3D-printed HFO in addition to a task-oriented approach and homework program has no beneficial effect on muscle force and manual dexterity in stroke survivors [40].

*Orthoses for joint contractures*. As no trials investigated the effectiveness of 3D-printed orthoses for joint contractures, conclusions cannot be made for this hand condition.

*Orthoses for pain*. One RCT of poor methodological quality showed that 3D-printed WHOs have no beneficial effect on pain reduction and functionality compared with prefabricated WHOs. There was a limited positive effect on satisfaction due to the small size of the 3D-printed WHO, snug fit and design that enabled water drainage [25].

### Gaps in knowledge

There were several gaps of knowledge identified. With regard to the outcomes on hand function, there is some evidence on the effectiveness of 3D-printed orthoses for forearm fractures and spasticity, but not for other hand conditions. Also, functionality as an outcome was scarcely investigated, indicating a knowledge gap of 3D-printed orthoses on performance benefits. Additionally, there is a knowledge gap on costs and production time of 3D-printed orthoses. Only few studies investigated adverse events, which are important to discover with regard to the practical utility of 3D-printed orthoses. Furthermore, there is a knowledge gap regarding the long-term effectiveness of 3D-printed orthosis, as the maximum follow up was 3 months. Assessing the long-term effectiveness is especially relevant for persons with chronic hand conditions, since they usually wear orthoses permanently. Lastly, since only four of 17 studies were controlled [25, 26, 33, 40], it can be concluded that there is a lack of good quality randomized controlled trials on the effectiveness of 3D-printed orthoses compared with conventional options to judge their added value on all outcomes of relevance.

### Limitations

Although we thoroughly followed the PRISMA-ScR checklist [27], there are some limitations that need to be addressed, such as the lack of searching for grey literature and the restriction to articles published only in English language. By excluding two RCTs published in Chinese and a case series in Portuguese [47–49], we may have omitted relevant findings.

## Conclusion

In this scoping review, seventeen studies on the effectiveness of 3D-printed orthoses for traumatic and chronic hand conditions were mapped and summarized. There is a clear need for high-quality controlled clinical trials to thoroughly investigate patient-related outcomes like hand function, functionality, satisfaction and adverse events using validated tools. Besides, an accurate analysis of production time and costs is needed to determine if 3D-printed hand orthoses may be integrated into clinical practice.

## Supporting information

S1 Appendix Pubmed search strategy. (DOCX)

S1 File Scoping review protocol. (PDF)

S1 Checklist PRISMA-ScR checklist. (PDF)
