# Supplementary material for: Effectiveness of 3D-printed orthoses for traumatic and chronic hand conditions: A scoping review
Source: PLoS One. 2021 Nov 18;16(11):e0260271. doi: 10.1371/journal.pone.0260271 (PMC8601455; doi:10.1371/journal.pone.0260271)
Supplement: S1 Appendix — (DOCX) [file pone.0260271.s001.docx]

**S1. APPENDIX - PubMed search strategy**

| **#** | **Searches** |
| --- | --- |
| **1** | “3d print*” |
| **2** | “3 dimensional print*” |
| **3** | “Three dimensional print*” |
| **4** | “Additive manufactur*” |
| **5** | “Additive fabricat*” |
| **6** | “Additive process*” |
| **7** | “Additive technique*” |
| **8** | “Freeform fabricat*” |
| **9** | “Selective Laser Sinter*” |
| **10** | “Fused deposition model*” |
| **11** | “Laminated object manufactur*” |
| **12** | “Layer Manufactur*” |
| **13** | “Rapid prototyp*” |
| **14** | “Direct Metal Laser Sinter*” |
| **15** | “Selective Laser Melt*” |
| **16** | Stereolithography |
| **17** | CAD-CAM |
| **18** | “Fused Filament Fabricat*” |
| **19** | 1 or 2 or 3 or 4 or 5 or 6 or 7 or 8 or 9 or 10 or 11 or 12 or 13 or 14 or 15 or 16 or 17 or 18 |
| **20** | "upper extremity" [MeSH] |
| **21** | Arm |
| **22** | Forearm |
| **23** | Hand |
| **24** | Wrist |
| **25** | Thumb |
| **26** | Finger |
| **27** | 20 or 21 or 23 or 24 or 25 or 26 |
| **28** | Orthosis |
| **29** | Orthoses |
| **30** | Brace |
| **31** | Splint |
| **32** | Cast |
| **33** | 28 or 29 or 30 or 31 or 32 |
| **34** | 19 and 27 and 33 |
