# Supplementary material for: Effectiveness of 3D-printed orthoses for traumatic and chronic hand conditions: A scoping review
Source: PLoS One. 2021 Nov 18;16(11):e0260271. doi: 10.1371/journal.pone.0260271 (PMC8601455; doi:10.1371/journal.pone.0260271)
Supplement: S1 File — (PDF) [file pone.0260271.s002.pdf]

Effectiveness of 3D-printed orthoses for traumatic and chronic hand conditions: a scoping review protocol

Protocol information

Contributors*

TAM Oud[1]

E Lazzari[2]

HJH Gijsbers[1]

M Gobbo[2,3]

F Nollet[1]

MA Brehm[1]

1. Amsterdam UMC, University of Amsterdam, Rehabilitation Medicine, Amsterdam Movement Sciences, Meibergdreef 9, Amsterdam, the Netherlands
2. Laboratory of Clinical Integrative Physiology, University of Brescia, Brescia, Italy
3. Department of Clinical and Experimental Sciences, University of Brescia, Brescia, Italy

Contact person

TAM Oud

e-mail: t.a.oud@amsterdamumc.nl

This protocol was drafted using the JBI methodology guidance for scoping reviews (JBI Reviewer's Manual)

* TAM Oud and E Lazzari contributed equally to this work as first authors.

INTRODUCTION

Hand function is important for the performance of activities. An injury to the underlying structures of the hand (including the wrist and fingers), either due to trauma or caused by neuro-musculoskeletal disorders (i.e. traumatic and chronic hand conditions) may lead to hand impairments like bone discontinuity, joint deformity, contractures, muscle weakness, spasticity, loss of sensibility, and/or pain.[1-4] These impairments may limit a person's ability in performing activities of daily living like eating, dressing and writing, as well as work- and leisure-related activities.[3-6] Accordingly, this can seriously impact on participation and quality of life.[5, 7, 8]

Orthoses, including casts, are commonly used in the treatment of traumatic and chronic hand conditions.[9-11] An orthosis is a rigid or semi rigid device used for the purpose of support, alignment, prevention or correction of joint deformities, or to improve function or restrict motion of a movable body part.[12] For many centuries, plaster casts and, more recently, fiberglass casts have been used in the treatment of traumatic hand conditions.[13, 14] These casts are low cost, strong, and easy to apply[15], and positive outcomes on pain, joint range of motion, and muscle strength have been shown in distal radius fractures and ligament injuries.[14, 16] Chronic hand conditions are commonly treated with custom fabricated orthoses of sustainable materials such as resin, leather, silicone or polypropylene.[17] In people with arthritis and post stroke it has been shown that these orthoses can reduce impairments like pain, muscle weakness and spasticity, and increase the ability to use the affected hand in daily activities.[18, 19]

Despite the reported benefits of conventional casts and custom fabricated orthoses, complications and discomfort have also been reported, including skin lesions (e.g. pressure sores, blisters), improper fit, sweating due to low breathability, heavy weight, bulkiness, and not being waterproof.[11, 15, 19] Since casts and custom fabricated

orthoses are handmade, the risks of complications and discomfort, especially skin lesions and improper fit largely depend on the practitioner's skills and experience.[11, 20] Furthermore, the manufacturing of custom fabricated orthoses is a labor intensive and time consuming process.[21]

In the last decade, the use of three-dimensional technology started to emerge in the field of orthotics, being a promising alternative to conventional orthoses. This technology involves three-dimensional scanning, modelling and printing, whereby materials are joined, layer by layer to manufacture 3D-printed orthoses.[20] So far, research into 3D-printed orthoses has mainly focused on the lower extremities, including two reviews on 3D-printed (ankle-)foot orthoses.[21, 22] These reviews concluded that the use of 3D printing to manufacture (ankle-)foot orthoses seems to have many potential benefits over conventional methods, in terms of improved comfort, fit and function. Furthermore, three-dimensional technology allows to eliminate several steps from the conventional manufacturing process of custom fabricated orthoses, and may improve efficiency by a shorter production time and lower costs.[20, 21, 23] While previous studies on the effects of 3D-printed orthoses for the upper extremities also indicated some of these benefits,[24-26] a synthesis of the results on the effectiveness of 3D-printed orthoses for the upper extremities, specifically with traumatic and chronic hand conditions is currently lacking.

A preliminary literature search conducted on September 4, 2020, in PubMed, JBI Evidence Synthesis and Open Science Framework registries identified that to date, no scoping or systematic reviews on 3D-printed hand orthoses have been performed and that none are currently underway. Also, the Cochrane Database of Systematic Reviews and the PROSPERO database were searched, revealing no systematic reviews on this topic. Since the use of 3D printing in manufacturing hand orthoses is

quite recent and the literature lacks high quality and homogeneous studies to perform a systematic review, we choose to perform a scoping review. The objective of this scoping review is to systematically map and summarize the research done on the effectiveness of 3D-printed orthoses for persons with traumatic and chronic hand conditions, as well as to identify any existing gaps in knowledge and needs for future research.

The scoping review will be conducted in accordance with the JBI methodology guidance for scoping reviews.[27]

METHODS

Eligibility criteria

Population

Eligible studies will include participants of any age with hand (including wrist and fingers) conditions due to trauma or chronic neurological, neuromuscular or musculoskeletal disorders.

Interventions

As treatment with 3D-printed orthoses for hand conditions is a relatively new concept and not much research has been conducted, the scoping review will include all types of 3D-printed hand orthoses, whether as a single intervention or combined with other interventions. Studies using orthoses with only small 3D-printed parts, and studies on 3D-printed prostheses and myoelectric orthoses will be excluded. In order to be

inclusive of any study that reports on 3D-printed hand orthoses, both studies involving any type of comparator and studies without a comparator will be included.

Outcome measures

Any outcome measure related to the effectiveness of 3D-printed hand orthoses, such as hand function, user satisfaction, adverse events, production time and costs will be included.

Types of studies

There will be no restrictions regarding publication year and study design, meaning that both RCTs and observational studies will be included. Studies will be restricted to the English language, and only full-text publications will be included. Ongoing studies, conference abstracts and posters will be excluded.

Search strategy

The first step will be to conduct a preliminary limited search of The Cochrane Library and PubMed databases, in order to identify the appropriate text words and index terms that will be used as keywords. A second literature search will be undertaken by one reviewer (EL) across the following electronic databases: The Cochrane Library (Cochrane Database of Systematic Reviews, Cochrane Central Register of Controlled Trials), PubMed, EMBASE, Web of Science, IEEE, CINAHL and PEDro. The search strategy will be formulated by two reviewers (EL and TO), combining the identified keywords and medical subject headings (MeSH) related to three-dimensional

technologies, anatomical body parts and interventions. No filters will be applied. The complete PubMed search strategy is outlined in the Appendix. This search strategy will be adapted for the other indexed databases. As a third step, the reference lists of the selected studies will be searched for additional relevant sources by the two reviewers. If needed, the authors of eligible studies will be contacted to ask for further information and resolve any uncertainties.

Selection of studies

The search results will be imported into Rayyan, a web-based literature screening program.[28] One reviewer (EL) will remove duplicates and will lead the process of study screening and selection supported by a second reviewer (TO). The two reviewers will independently screen the title and abstract of the search results using the predetermined eligibility criteria to in- or exclude the studies. Each excluded article will be labelled with an exclusion reason in Rayyan. Full-texts will be retrieved and evaluated if it is unclear whether the study meets the eligibility criteria. Conflicts regarding inclusion status will be resolved by discussion. If no consensus is achieved, a third reviewer (MB) will be consulted.

Data extraction

Each study will be charted by one reviewer (EL) using a data extraction table designed in Microsoft Excel. The charted data will be verified by a second reviewer (TO). After discussion, the data extraction table will be eventually updated and refined. Any refinements will be explained in the scoping review report. The following key study characteristics will be extracted: study design, subjects (sample size, age, and

diagnosis), intervention(s) (type of orthosis, frequency and duration of wearing, follow-up, and, if present, description of co-interventions). If disagreements will occur between reviewers, a third reviewer (MB) will be consulted.

Critical appraisal of studies

To provide a qualitative overview of the existing evidence, the randomized controlled trials and uncontrolled clinical trials included in this review will be critically appraised. The Modified Downs and Black checklist is chosen since it can be used to assess the methodological quality of randomized controlled studies, as well as non-randomized studies.[29] Prior to the critical appraisal, the reviewers (EL, TO, MB) will discuss the checklist's items to ensure the same interpretation. Two reviewers (EL and TO) will independently assess the studies. Disagreements will be resolved through discussion and consensus, if necessary with a third reviewer (MB).

RESULTS

Analysis of the evidence

First, the study selection process will be described. Second, an overview of the characteristics of the included studies will be provided. Third, the results of the methodological quality assessment will be shown. To meet the objectives of the review, data concerning the identified outcome measures will be mapped, summarizing the existing research findings. Furthermore, research gaps in the existing literature will be identified and recommendations for future research in this field will be made.

Presentation of the results

The study selection process of search results will be shown in a PRISMA flow diagram. The number of studies mapped to each characteristic will be summarized by numerical counts, and the data extraction table will be presented. Critical appraisal scores of the included studies will be tabulated. Outcome measure findings will be descriptively synthesised. If there will be availability of identical outcomes and sufficient homogeneity, data will be pooled and subgroup analysis will be carried out. Findings may be also mapped by a tabular presentation to provide an overview of the outcomes investigated in the included studies. Nevertheless, the development of the framework will be an iterative process that will be refined according to what will emerge while conducting the review.

Conflicts of interest

The contributors declare that there are no conflicts of interest.

Funding

The contributors receive no financial support for the research, future authorship, and/or publication of this scoping review.

REFERENCES

1. Gündüz OH BO. Chapter 8: Hand function in stroke. In: Duruöz MT (ed) *Hand Function: A Practical Guide to Assessment*. First ed. New York: Springer-Verlag, 2014, pp.107-114.

2. Videler AJ, van Dijk JP, Beelen A, et al. Motor axon loss is associated with hand dysfunction in Charcot-Marie-Tooth disease 1a. *Neurology* 2008; 71: 1254-1260. 2008/10/15. DOI: 10.1212/01.wnl.0000327643.05073.eb.

3. Altman R. Chapter 5: Hand function in osteoarthritis. In: Duruöz MT (ed) *Hand Function: A Practical Guide to Assessment*. First ed. New York: Springer-Verlag, 2014, pp.63-69.

4. Gustafsson M, Hagberg L and Holmefur M. Ten years follow-up of health and disability in people with acute traumatic hand injury: pain and cold sensitivity are long-standing problems. *J Hand Surg Eur Vol* 2011; 36: 590-598. 2011/05/20. DOI: 10.1177/1753193411408186.

5. Arwert H, Schut S, Boiten J, et al. Patient reported outcomes of hand function three years after stroke. *Top Stroke Rehabil* 2018; 25: 13-19. 2017/10/14. DOI: 10.1080/10749357.2017.1385232.

6. Eklund E, Svensson E and Hager-Ross C. Hand function and disability of the arm, shoulder and hand in Charcot-Marie-Tooth disease. *Disabil Rehabil* 2009; 31: 1955-1962. 2009/05/30. DOI: 10.1080/09638280902874170.

7. Rosberg HE, Carlsson KS and Dahlin LB. Prospective study of patients with injuries to the hand and forearm: costs, function, and general health. *Scand J Plast Reconstr Surg Hand Surg* 2005; 39: 360-369. 2005/11/22. DOI: 10.1080/02844310500340046.

8. Michon M, Maheu E and Berenbaum F. Assessing health-related quality of life in hand osteoarthritis: a literature review. *Ann Rheum Dis* 2011; 70: 921-928. 2011/03/15. DOI: 10.1136/ard.2010.131151.

9. Becker SJ, Bot AG, Curley SE, et al. A prospective randomized comparison of neoprene vs thermoplast hand-based thumb spica splinting for trapeziometacarpal arthrosis. *Osteoarthritis Cartilage* 2013; 21: 668-675. 2013/03/06. DOI: 10.1016/j.joca.2013.02.006.

10. Videler A, Eijffinger E, Nollet F, et al. A thumb opposition splint to improve manual dexterity and upper-limb functioning in Charcot-Marie-Tooth disease. *J Rehabil Med* 2012; 44: 249-253. 2012/03/01. DOI: 10.2340/16501977-0932.

11. Shirley ED, Maguire KJ, Mantica AL, et al. Alternatives to Traditional Cast Immobilization in Pediatric Patients. *J Am Acad Orthop Surg* 2020; 28: e20-e27. 2019/07/11. DOI: 10.5435/JAAOS-D-18-00152.

12. Jacobs M and Coverdale J. Concepts of Orthotic Fundamentals. In: Jacobs M and Austin N (eds) *Orthotic Intervention for the Hand and Upper Extremity: Splinting Principles and Process*. Second ed. Baltimore, Philadelphia: Wolters Kluwer Health/Lippincott Williams & Wilkins, 2014, pp.2-25.

13. Szostakowski B, Smitham P and Khan WS. Plaster of Paris-Short History of Casting and Injured Limb Immobilzation. *Open Orthop J* 2017; 11: 291-296. 2017/06/02. DOI: 10.2174/1874325001711010291.

14. Toon DH, Premchand RAX, Sim J, et al. Outcomes and financial implications of intra-articular distal radius fractures: a comparative study of open reduction internal fixation (ORIF) with volar locking plates versus nonoperative management. *J Orthop Traumatol* 2017; 18: 229-234. 2017/02/06. DOI: 10.1007/s10195-016-0441-8.

15. Graham J, Wang M, Frizzell K, et al. Conventional vs 3-Dimensional Printed Cast Wear Comfort. *Hand (N Y)* 2018: 1558944718795291. 2018/08/28. DOI: 10.1177/1558944718795291.

16. Gaston RG and Lourie GM. Radial collateral ligament injury of the index metacarpophalangeal joint: an underreported but important injury. *J Hand Surg Am* 2006; 31: 1355-1361. 2006/10/10. DOI: 10.1016/j.jhsa.2006.05.015.

17. Supan TJ. Chapter 4: Principles of fabrication. In: Hsu JD MJ, Fisk R. (ed) *AAOS Atlas of Orthoses and Assistive Devices*. 4th ed. Philadelphia: Mosby Elsevier, 2008, pp.53-59.

18. Haskett S, Backman C, Porter B, et al. A crossover trial of custom-made and commercially available wrist splints in adults with inflammatory arthritis. *Arthritis Rheum* 2004; 51: 792-799. 2004/10/13. DOI: 10.1002/art.20699.

19. Andringa AS, Van de Port IG and Meijer JW. Tolerance and effectiveness of a new dynamic hand-wrist orthosis in chronic stroke patients. *NeuroRehabilitation* 2013; 33: 225-231. 2013/08/21. DOI: 10.3233/NRE-130949.

20. Barrios-Muriel J, Romero-Sanchez F, Alonso-Sanchez FJ, et al. Advances in Orthotic and Prosthetic Manufacturing: A Technology Review. *Materials (Basel)* 2020; 13 2020/01/16. DOI: 10.3390/ma13020295.

21. Chen R. JY-a, Wensman J., Shih A. Additive manufacturing of custom orthoses and prostheses - a review. *Additive manufacturing* 2015; 12: 77-89.

22. Wojciechowski E, Chang AY, Balassone D, et al. Feasibility of designing, manufacturing and delivering 3D printed ankle-foot orthoses: a systematic review. *J Foot Ankle Res* 2019; 12: 11. 2019/02/19. DOI: 10.1186/s13047-019-0321-6.

23. Cha YH, Lee KH, Ryu HJ, et al. Ankle-Foot Orthosis Made by 3D Printing Technique and Automated Design Software. *Appl Bionics Biomech* 2017; 2017: 9610468. 2017/08/23. DOI: 10.1155/2017/9610468.

24. Guida P, Casaburi A, Busiello T, et al. An alternative to plaster cast treatment in a pediatric trauma center using the CAD/CAM technology to manufacture customized three-dimensional-printed orthoses in a totally hospital context: a feasibility study. *J Pediatr Orthop B* 2019; 28: 248-255. 2019/02/16. DOI: 10.1097/BPB.0000000000000589.

25. Kim SJ, Kim SJ, Cha YH, et al. Effect of personalized wrist orthosis for wrist pain with three-dimensional scanning and printing technique: A preliminary, randomized, controlled, open-label study. *Prosthet Orthot Int* 2018; 42: 636-643. 2018/07/17. DOI: 10.1177/0309364618785725.

26. Zheng Y, Liu G, Yu L, et al. Effects of a 3D-printed orthosis compared to a low-temperature thermoplastic plate orthosis on wrist flexor spasticity in chronic hemiparetic stroke patients: a randomized controlled trial. *Clin Rehabil* 2020; 34: 194-204. 2019/11/07. DOI: 10.1177/0269215519885174.

27. Moher D, Schulz KF, Simera I, et al. Guidance for developers of health research reporting guidelines. *PLoS Med* 2010; 7: e1000217. 2010/02/20. DOI: 10.1371/journal.pmed.1000217.

28. Ouzzani M, Hammady H, Fedorowicz Z, et al. Rayyan-a web and mobile app for systematic reviews. *Syst Rev* 2016; 5: 210. 2016/12/07. DOI: 10.1186/s13643-016-0384-4.

29. Trac MH, McArthur E, Jandoc R, et al. Macrolide antibiotics and the risk of ventricular arrhythmia in older adults. *CMAJ* 2016; 188: E120-E129. 2016/02/24. DOI: 10.1503/cmaj.150901.

APPENDIX

PubMed search strategy

#	Searches
**1**	3d print*
**2**	3 dimensional print*
**3**	Three dimensional print*
**4**	Additive manufactur*
**5**	Additive fabricat*
**6**	Additive process*
**7**	Additive technique*
**8**	Freeform fabricat*
**9**	Selective Laser Sinter*
**10**	Fused deposition model*
**11**	Laminated object manufactur*
**12**	Layer Manufactur*
**13**	Rapid prototyp*
**14**	Direct Metal Laser Sinter*
**15**	Selective Laser Melt*
**16**	Stereolithography
**17**	CAD-CAM
**18**	Fused Filament Fabricat*
**19**	1 or 2 or 3 or 4 or 5 or 6 or 7 or 8 or 9 or 10 or 11 or 12 or 13 or 14 or 15 or 16 or 17 or 18
**20**	"upper extremity" [MeSH]
**21**	Arm
**22**	Forearm
**23**	Hand
**24**	Wrist
**25**	Thumb

**26**	Finger
**27**	20 or 21 or 23 or 24 or 25 or 26
**28**	Orthosis
**29**	Orthoses
**30**	Brace
**31**	Splint
**32**	Cast
**33**	28 or 29 or 30 or 31 or 32
**34**	19 and 27 and 33
