# Supplementary material for: Effectiveness of 3D-printed orthoses for traumatic and chronic hand conditions: A scoping review
Source: PLoS One. 2021 Nov 18;16(11):e0260271. doi: 10.1371/journal.pone.0260271 (PMC8601455; doi:10.1371/journal.pone.0260271)
Supplement: Attachment — Submitted filename: Response to reviewers.docx [file pone.0260271.s004.docx]

­­

|  | | Amsterdam UMC, location AMC  Dept. of Rehabilitation Medicine  PO Box 22660  1100DD Amsterdam  The Netherlands  ☏ +31 20 5662615  ✉ t.a.oud@amsterdamumc.nl |
| --- | --- | --- |
|  | |  |
| Date: | July 21, 2021 |  |
| Subject: | Revised manuscript PONE-D-21-10493 | |

Dear Editor,

We acknowledge the reviewers for their valuable comments, which helped us to improve our manuscript. Please find enclosed the revised manuscript. A detailed response to the issues raised by the reviewers is provided in the appendix to this letter. Changes made in the manuscript are indicated by track changes. Also an unmarked version of the revised manuscript without tracked changes is attached. We hope the paper is now suitable for publication in PlosOne.

On behalf of the authors,

Yours sincerely,

Tanja Oud

APPENDIX - Reviewer comments

**Reviewer #1**

1. *“Scoping reviews require rigorous and transparent methods in their conduct to ensure that the results are trustworthy; It is recommended to consult papers like Arskey and O'malley and reporting guidelines for scoping reviews to organize methods and reporting.”*

Response: In 2015, the Joanna Briggs Institute (JBI) published a guidance document for the conduct of scoping reviews, which is based on the earlier work of Arksey and O’Malley (2005) and Levac and colleagues (2010). Besides a guidance for conducting a scoping review, the JBI also developed a reporting guideline for scoping reviews, the Preferred Reporting Items for Systematic Reviews and Meta-Analyses – Scoping Reviews (PRISMA-ScR) checklist [Tricco, 2018]. As described in the Methods section (page 6 lines 117-119), we followed the JBI guidelines and the PRISMA-ScR checklist for the conduct and reporting of our scoping review.

2. *“It is recommended that if the authors have a question addressing the feasibility, appropriateness, meaningfulness or effectiveness of a specific treatment or practice, then a systematic review is likely the most valid approach. This review has critical appraisal - a feature of systematic reviews. It seems a hybrid method.”*

Response: It is true that we included a critical appraisal, which is a feature of systematic reviews. However, for the conduct of our scoping review, we followed the JBI guidelines that state that also for scoping reviews there can be indications to identify the types of available evidence in a given field and to identify knowledge gaps. Also, the PRISMA-ScR checklist includes an optional item (item 12) ‘Critical Appraisal of Individual Sources of Evidence’. We decided to include this step in our scoping review, because in this way the results of the outcomes can be interpreted along with the knowledge about the methodological quality of the included studies. For clarification, we added this rationale in the ‘Critical appraisal of studies’ paragraph within the Methods section (page 9 lines 179-180).

3. *“Considering the aim of this study to know the effectiveness of 3D-printed orthoses for traumatic and chronic hand conditions (page 6-l 106), conducting a systematic review is more relevant than a scoping review.”*

Response: In the Introduction section (page 6 lines 109-111), we described the rationale for performing a scoping review instead of a systematic review. This rationale was supported by the review outcomes, as pooling the results of the outcomes would not be appropriate, because of the limited, heterogeneous, and mostly low/poor methodological quality of the studies.

*4. “Abstract: “In results: here is the only place that the authors linked the results from appraising the evidence and narrative description of the extracted papers.”*

Response: In the ‘Summary of main results’ paragraph of the Discussion section, we linked the results of the critical appraisal with the narrative description of the extracted papers (pages 28-30).

*5. “Introduction: The main aim of the scoping reviews is to describe the nature and extent of the published research, not synthesis the available evidence.”*

Response: We agree with the reviewer on this. In the last sentence of the introduction, we stated that our objective was to systematically map and summarize the published research on the effectiveness of 3D-printed orthoses for traumatic and chronic hand conditions, and to identify any existing gaps in knowledge and needs for future research. We didn’t include a synthesis of the available evidence.

*6.	“Search strategy: Please indicate your keywords in your search strategy.”*

Response: The specified search string for PubMed was included in ‘S1 Appendix’. For clarification, we added the topics of the keywords and MeSH terms in the ‘Search strategy’ paragraph of the Methods section (page 8, lines 151-152).

*7.	“There is nothing about your criteria for inclusion/exclusion in this section.”*

Response: The PRISMA-ScR checklist uses the subheading ‘Eligibility criteria’ to address the inclusion and exclusion criteria. We used the same subheading. The information on the inclusion and exclusion criteria can be found on page 7.

*8. “Critical appraisal of the included studies is not part of conducting a scoping review. However, despite using the appraisal tool, the authors did not use their appraisal results in their report.”*

Response: Please, see our response on comments 2 and 4.

*9.	“Page 10, line 204-5, Please do not use the abbreviations for the first time in your manuscript (RCT, NRCT)”*

Response: The abbreviations (RCT, NRS) were already introduced in the paragraph ‘Critical appraisal of studies’ in the Methods section (page 9 lines 180-181).

*10. “Table 1: What do you mean by chronic hand conditions?
The ready-made orthosis is not a known name for hand therapists; you can use custom-made or prefabricated. Why did you separate splint from orthosis? Both are the same.”*

Response: In the Introduction section, (page 4 lines 56-63), chronic hand conditions were explained. However, to be more clear about what we meant with chronic hand conditions, we modified the text. We agree with the reviewer that prefabricated is a better word then ready-made orthosis and that a splint is the same as an orthosis. Therefore, we replaced ready-made for prefabricated throughout the manuscript, and we changed splint into orthosis.

*11. “Critical appraisal is not part of scoping reviews. There is no information regarding the appraisal tool and how the authors used the extracted scores in their results.”*

Response: As explained in our response on comment 2, the most recent guidelines for conducting a scoping review include the option of a critical appraisal of the included studies. In the paragraph ‘Critical appraisal of the studies’ in the Methods section (page 9), we described that we used the Modified Downs and Black checklist as appraisal tool. In the paragraph ‘Results of critical appraisal’ (page 15), we presented the results of the critical appraisal and combined these results with the outcomes of the studies in the ‘Summary of main findings’ in the Discussion (page 28).

*12. “The authors used the narrative method for reporting their results. There is no connection between the results from appraisal and the narrative results based on the impairment.”*

Response: Please, see our response on comments 4 and 11.

*13.	“The reported results are not well organized, and the reader cannot link them to the original review questions and objectives.”*

Response: The objective of our scoping review was to systematically map and summarize the research done on the effectiveness of 3D-printed orthoses for traumatic and chronic hand conditions, and identify any existing gaps in knowledge and needs for future research. The JBI suggests to map out the gathered information in a logical, diagrammatic or tabular form, and/or in a descriptive form. We presented a Prisma-ScR flow diagram and four tables, each of them linking to our objective. We choose to describe the results of the outcomes as they represent the effectiveness of the 3D-printed orthosis. Furthermore, in the Discussion we pointed out several gaps in knowledge.

*14.	“There is no description for the table 3 and 4 in the manuscript.”*

Response: As suggested by the reviewer, we added a description for table 3 in the ‘Results of critical appraisal’ paragraph (page 15, line 237) and for table 4 in the ‘Synthesis of results’ paragraph (page 17, lines 254-255).

*15. “Discussion: Page 26, line 432; why the authors included all published in the last four years? If this was part of the inclusion criteria, it should be mentioned.”*

Response: We did not set an inclusion criterion for publication date. Yet, it appeared that all studies were published the last four years. This is a result, not an inclusion criteria.

*16. “Lines 439-450: There is no information about the effectiveness of the 3D print orthosis neither in the results nor in the discussion.”*

Response: In the paragraph ‘Synthesis of results’ in the Results section (page 17), we presented the identified outcomes related to the effectiveness of 3D-printed orthoses. These outcomes (hand function, functionality, satisfaction, production time, costs, adverse events) were further described and discussed in the Results and Discussion sections, and represent in fact the effectiveness of 3D-printed orthoses.

**----------------------------------------------------------------------------------------------**

**Reviewer #2**

1. *“This is a well written, methodologically robust review in an emerging area of orthotic practice.”*

Response: Thank you for the positive comment.

2. *“I find the terminology used to describe the population/conditions studied a little curious. Firstly it appears that the authors looked at wrist and hand conditions, rather than just hand. So this needs to be adjusted throughout the text. Secondly they selected studies on pretty much any wrist and hand condition but then split them into ‘traumatic’ and ‘chronic’. ‘Traumatic’ and ‘non-traumatic’ would be more accurate, but I am not sure why the distinction was made.”*

Response: For the readability of the manuscript, in the Introduction section and in the methods section, we defined that hand conditions include also the wrist and fingers, see page 4 lines 61-62 and page 7 line 128. Furthermore, we split the hand conditions into traumatic and chronic hand conditions to structure all the gathered information. A person who is more interested in using 3D-printed orthoses in traumatic hand conditions can focus on the results of this specific group, whereas a person interested in the use of 3D-printed orthoses for chronic hand conditions can directly read the information of this specific group. Lastly, we think that using ‘chronic’ hand conditions instead of ‘non-traumatic’ better expresses the fact that these conditions, caused by neuro-musculoskeletal disorders or long-lasting complaints resulting from traumatic hand conditions, will be permanent. Also, the term ‘non-traumatic’ would suggest that traumatic is the norm, while ‘chronic’ is more neutral encompassing a broad range of possible causes.

*3.	“The method and results are excellent.”*

Response: We appreciate this compliment.

*4.	“Discussion*: *There is a tendency to repeat the results eg the outcomes used and the results found. Try to avoid this. The summary of findings should be just that- a paragraph should cover it. The assessment of quality could make it clearer why the quality was poor. I imagine that blinding and concealment of allocation was issue, not just the unpowered sample sizes.”*

Response: As suggested by the reviewer, we reduced the amount of words in the ‘Summary of main results’ paragraph in the discussion (pages 28-30). Further, we agree with the reviewer on the reasons of the poor methodological quality of the studies and have specified this more in detail in the Results of critical appraisal (page 15).

5. *“The gaps in knowledge are a little generous”*

Response: We acknowledge the reviewer’s suggestion, and stated the gaps of knowledge somewhat more generous, specifically indicating the need for good quality randomized controlled trials on 3D-printed orthoses for all outcomes (pages 30-31).
