# Supplementary material for: Effectiveness of 3D-printed orthoses for traumatic and chronic hand conditions: A scoping review
Source: PLoS One. 2021 Nov 18;16(11):e0260271. doi: 10.1371/journal.pone.0260271 (PMC8601455; doi:10.1371/journal.pone.0260271)
Supplement: Attachment — Submitted filename: Response to reviewers_01.10.21.docx [file pone.0260271.s005.docx]

­­

|  | | Amsterdam UMC, location AMC  Dept. of Rehabilitation Medicine  PO Box 22660  1100DD Amsterdam  The Netherlands  ☏ +31 20 5662615  ✉ t.a.oud@amsterdamumc.nl |
| --- | --- | --- |
|  | |  |
| Date: | October 1, 2021 |  |
| Subject: | Revised manuscript PONE-D-21-10493R1 | |

Dear Editor,

We are happy that the reviewers approved the changes made to our revised manuscript entitled ‘*Effectiveness of 3D-printed orthoses for traumatic and chronic hand conditions: a scoping review’* (PONE-D-21-10493R1). Reviewer 1 had only one comment left, which is addressed in the appendix to this letter. Please find enclosed the revised manuscript. Changes made in the manuscript are indicated by track changes. Also an unmarked version of the revised manuscript without tracked changes is attached. We hope the paper is now suitable for publication in PLOS ONE.

On behalf of the authors,

Yours sincerely,

Tanja Oud

APPENDIX - Reviewer comments

**Reviewer #1**

*“I would like to see some details in their description chart about how the 3D printing was performed.”*

Response: As suggested by the reviewer, we inserted a column in table 2 (pages 13-15) with information on the 3D printing process. No or limited information was described in the included studies on who prescribed the 3D-printed orthoses and who fabricated the orthoses. However, we were able to obtain information of the way of geometry acquisition, the software used, type of 3D printing, and material used.

Furthermore, on page 11 line 223, we specified that information regarding the 3D printing process is presented in table 2.

---------------------------------------------------------

**Reviewer #2**

The reviewer indicated that all comments have been thoroughly addressed.
