# Supplementary material for: Effectiveness of 3D-printed orthoses for traumatic and chronic hand conditions: A scoping review
Source: PLoS One. 2021 Nov 18;16(11):e0260271. doi: 10.1371/journal.pone.0260271 (PMC8601455; doi:10.1371/journal.pone.0260271)
Supplement: Peer review history

# Peer review: Decision Letter 0

**DOI:** 10.1371/journal.pone.0260271.r001

**Authors:** Sebastian Grunt

30 Jun 2021

PONE-D-21-10493

Effectiveness of 3D-printed orthoses for traumatic and chronic hand conditions: a scoping review

PLOS ONE

Dear Dr. Oud,

Thank you for submitting your manuscript to PLOS ONE. After careful consideration, we feel that it has merit but does not fully meet PLOS ONE’s publication criteria as it currently stands. Therefore, we invite you to submit a revised version of the manuscript that addresses the points raised during the review process.

Please ensure that your decision is justified on PLOS ONE’s publication criteria and not, for example, on novelty or perceived impact.

For Lab, Study and Registered Report Protocols: These article types are not expected to include results but may include pilot data. 

Please submit your revised manuscript by Aug 14 2021 11:59PM. If you will need more time than this to complete your revisions, please reply to this message or contact the journal office at plosone@plos.org. When you're ready to submit your revision, log on to https://www.editorialmanager.com/pone/ and select the 'Submissions Needing Revision' folder to locate your manuscript file.

Please include the following items when submitting your revised manuscript:

- A rebuttal letter that responds to each point raised by the academic editor and reviewer(s). You should upload this letter as a separate file labeled 'Response to Reviewers'.
- A marked-up copy of your manuscript that highlights changes made to the original version. You should upload this as a separate file labeled 'Revised Manuscript with Track Changes'.
- An unmarked version of your revised paper without tracked changes. You should upload this as a separate file labeled 'Manuscript'.

If you would like to make changes to your financial disclosure, please include your updated statement in your cover letter. Guidelines for resubmitting your figure files are available below the reviewer comments at the end of this letter.

If applicable, we recommend that you deposit your laboratory protocols in protocols.io to enhance the reproducibility of your results. Protocols.io assigns your protocol its own identifier (DOI) so that it can be cited independently in the future. For instructions see: http://journals.plos.org/plosone/s/submission-guidelines#loc-laboratory-protocols. Additionally, PLOS ONE offers an option for publishing peer-reviewed Lab Protocol articles, which describe protocols hosted on protocols.io. Read more information on sharing protocols at https://plos.org/protocols?utm_medium=editorial-email&utm_source=authorletters&utm_campaign=protocols.

We look forward to receiving your revised manuscript.

Kind regards,

Sebastian Grunt

Academic Editor

PLOS ONE

Journal requirements:

When submitting your revision, we need you to address these additional requirements.

1. Please ensure that your manuscript meets PLOS ONE's style requirements, including those for file naming. The PLOS ONE style templates can be found at

https://journals.plos.org/plosone/s/file?id=wjVg/PLOSOne_formatting_sample_main_body.pdf and

https://journals.plos.org/plosone/s/file?id=ba62/PLOSOne_formatting_sample_title_authors_affiliations.pdf.

Additional Editor Comments (if provided):

[Note: HTML markup is below. Please do not edit.]

Reviewers' comments:

Reviewer's Responses to Questions

**Comments to the Author**

1. Is the manuscript technically sound, and do the data support the conclusions?

The manuscript must describe a technically sound piece of scientific research with data that supports the conclusions. Experiments must have been conducted rigorously, with appropriate controls, replication, and sample sizes. The conclusions must be drawn appropriately based on the data presented. 

Reviewer #1: Partly

Reviewer #2: Yes

**********

2. Has the statistical analysis been performed appropriately and rigorously? 

Reviewer #1: No

Reviewer #2: N/A

**********

3. Have the authors made all data underlying the findings in their manuscript fully available?

The PLOS Data policy requires authors to make all data underlying the findings described in their manuscript fully available without restriction, with rare exception (please refer to the Data Availability Statement in the manuscript PDF file). The data should be provided as part of the manuscript or its supporting information, or deposited to a public repository. For example, in addition to summary statistics, the data points behind means, medians and variance measures should be available. If there are restrictions on publicly sharing data—e.g. participant privacy or use of data from a third party—those must be specified.

Reviewer #1: Yes

Reviewer #2: Yes

**********

4. Is the manuscript presented in an intelligible fashion and written in standard English?

PLOS ONE does not copyedit accepted manuscripts, so the language in submitted articles must be clear, correct, and unambiguous. Any typographical or grammatical errors should be corrected at revision, so please note any specific errors here.

Reviewer #1: Yes

Reviewer #2: Yes

**********

5. Review Comments to the Author

Please use the space provided to explain your answers to the questions above. You may also include additional comments for the author, including concerns about dual publication, research ethics, or publication ethics. (Please upload your review as an attachment if it exceeds 20,000 characters)

Reviewer #1: Effectiveness of 3D-printed orthoses for traumatic and chronic hand conditions: a scoping review

Thank you for the opportunity to review this paper on systematically mapping and summarizing the research done on the effectiveness of 3D-printed orthoses for traumatic and chronic hand conditions. Scoping reviews require rigorous and transparent methods in their conduct to ensure that the results are trustworthy; It is recommended you consult papers like arskey and o'malley and reporting guidelines for scoping reviews to organize methods and reporting. It is recommended that if the authors have a question addressing the feasibility, appropriateness, meaningfulness or effectiveness of a specific treatment or practice, then a systematic review is likely the most valid approach. (Pearson A, Wiechula R, Court A, Lockwood C. The JBI model of evidence-based healthcare. International Journal of Evidence-Based Healthcare. 2005;3(8):207–15.). This review has critical appraisal - a feature of systematic reviews. It seems a hybrid method.

Considering the aim of this study to know the effectiveness of 3D-printed orthoses for traumatic and chronic hand conditions (page 6-l 106), conducting a systematic review is more relevant than a scoping review. However, if you want to char the approaches and methods used in the research - a scoping review is appropriate. A scoping review ften is performed to see fi the state of evidnence is ready for a systematic review- which i might suggest it is not.

Abstract

In results: here is the only place that the authors linked the results from appraising the evidence and narrative description of the extracted papers.

Introduction;

The main aim of the scoping reviews is to describe the nature and extent of the published research, not synthesis the available evidence.

Search strategy:

Please indicate your keywords in your search strategy.

There is nothing about your criteria for inclusion/exclusion in this section.

Critical appraisal of the included studies is not part of conducting a scoping review. You cannot map the current status of evidence by excluding low-quality studies. However, despite using the appraisal tool, the authors did not use their appraisal results in their report.

Page 10, line 204-5, Please do not use the abbreviations for the first time in your manuscript (RCT, NRCT)

Table 1: What do you mean by chronic hand conditions?

Contractures are chronic deformities; why you separated these?

The ready-made orthosis is not a known name for hand therapists; you can use custom-made or prefabricated. Why did you separate splint from orthosis? Both are the same.

Critical appraisal is not part of scoping reviews. Look at: A scoping review on the conduct and reporting of scoping reviews. It was possible to conduct a systematic review and subgroup analysis with four RCTs based on the outcomes.

There is no information regarding the appraisal tool and how the authors used the extracted scores in their results.

The authors used the narrative method for reporting their results. There is no connection between the results from appraisal and the narrative results based on the impairment.

The reported results are not well organized, and the reader cannot link them to the original review questions and objectives.

There is no description for the table 3 and 4 in the manuscript.

Discussion

Page 26, line 432; why the authors included all published in the last four years? If this was part of the inclusion criteria, it should be mentioned.

Lines 439-450: all the discussion is about the outcomes. There is no information about the effectiveness of the 3D print orthosis neither in the results nor in the discussion.

Reviewer #2: This is a well written, methodologically robust review in an emrging area of orthotic practice.

A few comments

I find the terminology used to describe the population/conditions studied a little curious. Firstly it appears that the authors looked at wrist and hand conditions, rather than just hand. SO this needs to be adjusted throughout the text. Secondly they selected studies on pretty much any wrist and hand condition but then split them into ‘traumatic’ and ‘chronic’. ‘Traumatic’ and ‘non-traumatic’ would be more accurate, but I am not sure why the distinction was made. It would be easier to get an overview of the state of the evidence if all studies were combined and described according to the problems they aimed to address.

The method and results are excellent.

Discussion- there is a tendency to repeat the results eg the outcomes used and the results found. Try to avoid this. The summary of findings should be just that- a paragraph should cover it. What you found was low quality evidence from a small number of studies, mainly assessing WHO on chronic conditions. The studies of traumatic injuries (all wrist fractures) suggested an overall reduction in pain; disability and AE (compared to plaster casts) and that patients were satisfied. However it was unclear how much of this improvement was due to healing of the fracture, rather than the type of orthosis/cast. The findings for non-traumatic conditions were mixed and no conclusions could be drawn.

The assessment of quality could make it clearer why the quality was poor. I imagine that blinding and concealment of allocation was issue, not just the unpowered sample sizes.

The gaps in knowledge a little generous – there is nothing definitive about the evidence so far, so many more gaps remain! It is reasonable to highlight the lack of work considering the effects on function/disability but there also needs to be good quality randomised controlled trials for all other outcomes too! Good to see satisfaction and adverse events highlighted, but all studies need to be comparison with current practice. The bottom line is to work out what advantage/improvement a 3D-printed orthosis has over what is currently available- if any.

**********

6. PLOS authors have the option to publish the peer review history of their article (what does this mean?). If published, this will include your full peer review and any attached files.

If you choose “no”, your identity will remain anonymous but your review may still be made public.

**Do you want your identity to be public for this peer review?** For information about this choice, including consent withdrawal, please see our Privacy Policy.

Reviewer #1: No

Reviewer #2: No

[NOTE: If reviewer comments were submitted as an attachment file, they will be attached to this email and accessible via the submission site. Please log into your account, locate the manuscript record, and check for the action link "View Attachments". If this link does not appear, there are no attachment files.]

While revising your submission, please upload your figure files to the Preflight Analysis and Conversion Engine (PACE) digital diagnostic tool, https://pacev2.apexcovantage.com/. PACE helps ensure that figures meet PLOS requirements. To use PACE, you must first register as a user. Registration is free. Then, login and navigate to the UPLOAD tab, where you will find detailed instructions on how to use the tool. If you encounter any issues or have any questions when using PACE, please email PLOS at figures@plos.org. Please note that Supporting Information files do not need this step.

# Author comment: Author response to Decision Letter 0

**DOI:** 10.1371/journal.pone.0260271.r002

21 Jul 2021

Reviewer #1

1. “Scoping reviews require rigorous and transparent methods in their conduct to ensure that the results are trustworthy; It is recommended to consult papers like Arskey and O'malley and reporting guidelines for scoping reviews to organize methods and reporting.”

Response: In 2015, the Joanna Briggs Institute (JBI) published a guidance document for the conduct of scoping reviews, which is based on the earlier work of Arksey and O’Malley (2005) and Levac and colleagues (2010). Besides a guidance for conducting a scoping review, the JBI also developed a reporting guideline for scoping reviews, the Preferred Reporting Items for Systematic Reviews and Meta-Analyses – Scoping Reviews (PRISMA-ScR) checklist [Tricco, 2018]. As described in the Methods section (page 6 lines 117-119), we followed the JBI guidelines and the PRISMA-ScR checklist for the conduct and reporting of our scoping review.

2. “It is recommended that if the authors have a question addressing the feasibility, appropriateness, meaningfulness or effectiveness of a specific treatment or practice, then a systematic review is likely the most valid approach. This review has critical appraisal - a feature of systematic reviews. It seems a hybrid method.”

Response: It is true that we included a critical appraisal, which is a feature of systematic reviews. However, for the conduct of our scoping review, we followed the JBI guidelines that state that also for scoping reviews there can be indications to identify the types of available evidence in a given field and to identify knowledge gaps. Also, the PRISMA-ScR checklist includes an optional item (item 12) ‘Critical Appraisal of Individual Sources of Evidence’. We decided to include this step in our scoping review, because in this way the results of the outcomes can be interpreted along with the knowledge about the methodological quality of the included studies. For clarification, we added this rationale in the ‘Critical appraisal of studies’ paragraph within the Methods section (page 9 lines 179-180).

3. “Considering the aim of this study to know the effectiveness of 3D-printed orthoses for traumatic and chronic hand conditions (page 6-l 106), conducting a systematic review is more relevant than a scoping review.”

Response: In the Introduction section (page 6 lines 109-111), we described the rationale for performing a scoping review instead of a systematic review. This rationale was supported by the review outcomes, as pooling the results of the outcomes would not be appropriate, because of the limited, heterogeneous, and mostly low/poor methodological quality of the studies.

4. “Abstract: “In results: here is the only place that the authors linked the results from appraising the evidence and narrative description of the extracted papers.”

Response: In the ‘Summary of main results’ paragraph of the Discussion section, we linked the results of the critical appraisal with the narrative description of the extracted papers (pages 28-30). 

5. “Introduction: The main aim of the scoping reviews is to describe the nature and extent of the published research, not synthesis the available evidence.”

Response: We agree with the reviewer on this. In the last sentence of the introduction, we stated that our objective was to systematically map and summarize the published research on the effectiveness of 3D-printed orthoses for traumatic and chronic hand conditions, and to identify any existing gaps in knowledge and needs for future research. We didn’t include a synthesis of the available evidence.

6. “Search strategy: Please indicate your keywords in your search strategy.”

Response: The specified search string for PubMed was included in ‘S1 Appendix’. For clarification, we added the topics of the keywords and MeSH terms in the ‘Search strategy’ paragraph of the Methods section (page 8, lines 151-152).

7. “There is nothing about your criteria for inclusion/exclusion in this section.”

Response: The PRISMA-ScR checklist uses the subheading ‘Eligibility criteria’ to address the inclusion and exclusion criteria. We used the same subheading. The information on the inclusion and exclusion criteria can be found on page 7.

8. “Critical appraisal of the included studies is not part of conducting a scoping review. However, despite using the appraisal tool, the authors did not use their appraisal results in their report.”

Response: Please, see our response on comments 2 and 4.

9. “Page 10, line 204-5, Please do not use the abbreviations for the first time in your manuscript (RCT, NRCT)”

Response: The abbreviations (RCT, NRS) were already introduced in the paragraph ‘Critical appraisal of studies’ in the Methods section (page 9 lines 180-181).

10. “Table 1: What do you mean by chronic hand conditions?

The ready-made orthosis is not a known name for hand therapists; you can use custom-made or prefabricated. Why did you separate splint from orthosis? Both are the same.”

Response: In the Introduction section, (page 4 lines 56-63), chronic hand conditions were explained. However, to be more clear about what we meant with chronic hand conditions, we modified the text. We agree with the reviewer that prefabricated is a better word then ready-made orthosis and that a splint is the same as an orthosis. Therefore, we replaced ready-made for prefabricated throughout the manuscript, and we changed splint into orthosis.

11. “Critical appraisal is not part of scoping reviews. There is no information regarding the appraisal tool and how the authors used the extracted scores in their results.”

Response: As explained in our response on comment 2, the most recent guidelines for conducting a scoping review include the option of a critical appraisal of the included studies. In the paragraph ‘Critical appraisal of the studies’ in the Methods section (page 9), we described that we used the Modified Downs and Black checklist as appraisal tool. In the paragraph ‘Results of critical appraisal’ (page 15), we presented the results of the critical appraisal and combined these results with the outcomes of the studies in the ‘Summary of main findings’ in the Discussion (page 28).

12. “The authors used the narrative method for reporting their results. There is no connection between the results from appraisal and the narrative results based on the impairment.”

Response: Please, see our response on comments 4 and 11.

13. “The reported results are not well organized, and the reader cannot link them to the original review questions and objectives.”

Response: The objective of our scoping review was to systematically map and summarize the research done on the effectiveness of 3D-printed orthoses for traumatic and chronic hand conditions, and identify any existing gaps in knowledge and needs for future research. The JBI suggests to map out the gathered information in a logical, diagrammatic or tabular form, and/or in a descriptive form. We presented a Prisma-ScR flow diagram and four tables, each of them linking to our objective. We choose to describe the results of the outcomes as they represent the effectiveness of the 3D-printed orthosis. Furthermore, in the Discussion we pointed out several gaps in knowledge.

14. “There is no description for the table 3 and 4 in the manuscript.”

Response: As suggested by the reviewer, we added a description for table 3 in the ‘Results of critical appraisal’ paragraph (page 15, line 237) and for table 4 in the ‘Synthesis of results’ paragraph (page 17, lines 254-255).

15. “Discussion: Page 26, line 432; why the authors included all published in the last four years? If this was part of the inclusion criteria, it should be mentioned.” 

Response: We did not set an inclusion criterion for publication date. Yet, it appeared that all studies were published the last four years. This is a result, not an inclusion criteria.

16. “Lines 439-450: There is no information about the effectiveness of the 3D print orthosis neither in the results nor in the discussion.”

Response: In the paragraph ‘Synthesis of results’ in the Results section (page 17), we presented the identified outcomes related to the effectiveness of 3D-printed orthoses. These outcomes (hand function, functionality, satisfaction, production time, costs, adverse events) were further described and discussed in the Results and Discussion sections, and represent in fact the effectiveness of 3D-printed orthoses.

----------------------------------------------------------------------------------------------

Reviewer #2

1. “This is a well written, methodologically robust review in an emerging area of orthotic practice.”

Response: Thank you for the positive comment.

2. “I find the terminology used to describe the population/conditions studied a little curious. Firstly it appears that the authors looked at wrist and hand conditions, rather than just hand. So this needs to be adjusted throughout the text. Secondly they selected studies on pretty much any wrist and hand condition but then split them into ‘traumatic’ and ‘chronic’. ‘Traumatic’ and ‘non-traumatic’ would be more accurate, but I am not sure why the distinction was made.”

Response: For the readability of the manuscript, in the Introduction section and in the methods section, we defined that hand conditions include also the wrist and fingers, see page 4 lines 61-62 and page 7 line 128. Furthermore, we split the hand conditions into traumatic and chronic hand conditions to structure all the gathered information. A person who is more interested in using 3D-printed orthoses in traumatic hand conditions can focus on the results of this specific group, whereas a person interested in the use of 3D-printed orthoses for chronic hand conditions can directly read the information of this specific group. Lastly, we think that using ‘chronic’ hand conditions instead of ‘non-traumatic’ better expresses the fact that these conditions, caused by neuro-musculoskeletal disorders or long-lasting complaints resulting from traumatic hand conditions, will be permanent. Also, the term ‘non-traumatic’ would suggest that traumatic is the norm, while ‘chronic’ is more neutral encompassing a broad range of possible causes. 

3. “The method and results are excellent.”

Response: We appreciate this compliment.

4. “Discussion: There is a tendency to repeat the results eg the outcomes used and the results found. Try to avoid this. The summary of findings should be just that- a paragraph should cover it. The assessment of quality could make it clearer why the quality was poor. I imagine that blinding and concealment of allocation was issue, not just the unpowered sample sizes.”

Response: As suggested by the reviewer, we reduced the amount of words in the ‘Summary of main results’ paragraph in the discussion (pages 28-30). Further, we agree with the reviewer on the reasons of the poor methodological quality of the studies and have specified this more in detail in the Results of critical appraisal (page 15).

5. “The gaps in knowledge are a little generous”

Response: We acknowledge the reviewer’s suggestion, and stated the gaps of knowledge somewhat more generous, specifically indicating the need for good quality randomized controlled trials on 3D-printed orthoses for all outcomes (pages 30-31).

Attachment Submitted filename: Response to reviewers.docx Click here for additional data file.

# Peer review: Decision Letter 1

**DOI:** 10.1371/journal.pone.0260271.r003

**Authors:** David Benjamin Lumenta

23 Sep 2021

PONE-D-21-10493R1Effectiveness of 3D-printed orthoses for traumatic and chronic hand conditions: a scoping reviewPLOS ONE

Dear Dr. Oud,

Thank you for submitting your manuscript to PLOS ONE. After careful consideration, we feel that it has merit but does not fully meet PLOS ONE’s publication criteria as it currently stands. Therefore, we invite you to submit a revised version of the manuscript that addresses the points raised during the review process.

Please submit your revised manuscript by Nov 07 2021 11:59PM. If you will need more time than this to complete your revisions, please reply to this message or contact the journal office at plosone@plos.org. When you're ready to submit your revision, log on to https://www.editorialmanager.com/pone/ and select the 'Submissions Needing Revision' folder to locate your manuscript file.

Please include the following items when submitting your revised manuscript:

- A rebuttal letter that responds to each point raised by the academic editor and reviewer(s). You should upload this letter as a separate file labeled 'Response to Reviewers'.
- A marked-up copy of your manuscript that highlights changes made to the original version. You should upload this as a separate file labeled 'Revised Manuscript with Track Changes'.
- An unmarked version of your revised paper without tracked changes. You should upload this as a separate file labeled 'Manuscript'.

If you would like to make changes to your financial disclosure, please include your updated statement in your cover letter. Guidelines for resubmitting your figure files are available below the reviewer comments at the end of this letter.

If applicable, we recommend that you deposit your laboratory protocols in protocols.io to enhance the reproducibility of your results. Protocols.io assigns your protocol its own identifier (DOI) so that it can be cited independently in the future. For instructions see: https://journals.plos.org/plosone/s/submission-guidelines#loc-laboratory-protocols. Additionally, PLOS ONE offers an option for publishing peer-reviewed Lab Protocol articles, which describe protocols hosted on protocols.io. Read more information on sharing protocols at https://plos.org/protocols?utm_medium=editorial-email&utm_source=authorletters&utm_campaign=protocols.

We look forward to receiving your revised manuscript.

Kind regards,

David Benjamin Lumenta, MD PhD

Academic Editor

PLOS ONE

Journal Requirements:

Please review your reference list to ensure that it is complete and correct. If you have cited papers that have been retracted, please include the rationale for doing so in the manuscript text, or remove these references and replace them with relevant current references. Any changes to the reference list should be mentioned in the rebuttal letter that accompanies your revised manuscript. If you need to cite a retracted article, indicate the article’s retracted status in the References list and also include a citation and full reference for the retraction notice.

Additional Editor Comments (if provided):

[Note: HTML markup is below. Please do not edit.]

Reviewers' comments:

Reviewer's Responses to Questions

**Comments to the Author**

1. If the authors have adequately addressed your comments raised in a previous round of review and you feel that this manuscript is now acceptable for publication, you may indicate that here to bypass the “Comments to the Author” section, enter your conflict of interest statement in the “Confidential to Editor” section, and submit your "Accept" recommendation.

Reviewer #1: (No Response)

Reviewer #2: All comments have been addressed

**********

2. Is the manuscript technically sound, and do the data support the conclusions?

The manuscript must describe a technically sound piece of scientific research with data that supports the conclusions. Experiments must have been conducted rigorously, with appropriate controls, replication, and sample sizes. The conclusions must be drawn appropriately based on the data presented. 

Reviewer #1: Yes

Reviewer #2: Yes

**********

3. Has the statistical analysis been performed appropriately and rigorously? 

Reviewer #1: Yes

Reviewer #2: N/A

**********

4. Have the authors made all data underlying the findings in their manuscript fully available?

The PLOS Data policy requires authors to make all data underlying the findings described in their manuscript fully available without restriction, with rare exception (please refer to the Data Availability Statement in the manuscript PDF file). The data should be provided as part of the manuscript or its supporting information, or deposited to a public repository. For example, in addition to summary statistics, the data points behind means, medians and variance measures should be available. If there are restrictions on publicly sharing data—e.g. participant privacy or use of data from a third party—those must be specified.

Reviewer #1: Yes

Reviewer #2: Yes

**********

5. Is the manuscript presented in an intelligible fashion and written in standard English?

PLOS ONE does not copyedit accepted manuscripts, so the language in submitted articles must be clear, correct, and unambiguous. Any typographical or grammatical errors should be corrected at revision, so please note any specific errors here.

Reviewer #1: Yes

Reviewer #2: Yes

**********

6. Review Comments to the Author

Please use the space provided to explain your answers to the questions above. You may also include additional comments for the author, including concerns about dual publication, research ethics, or publication ethics. (Please upload your review as an attachment if it exceeds 20,000 characters)

Reviewer #1: i think this acceptable based on response to prior review . I would like to see some details in their description chart about how the 3D printing was performed. It seems like one of the main point of this paper is that 3D splints made by a machine based on inputs from a human being (not splints made directly and custom fit by a therapist) were used so it would be important to have some details on not just for whom and what clinical condition ( which is in the chart); but also by whom, who did prescription, fitting, what device printed etc. There is a good description of clinical use but limited description of the 3D printing process and how it is different from typical practice which seems important given the topic. Otherwise looks great.

Reviewer #2: The authors have thoroughly addressed my comments in the original review. Apparently there is a minimum word count I have to reach in order to submit htis review. So here is some padding. How silly

**********

7. PLOS authors have the option to publish the peer review history of their article (what does this mean?). If published, this will include your full peer review and any attached files.

If you choose “no”, your identity will remain anonymous but your review may still be made public.

**Do you want your identity to be public for this peer review?** For information about this choice, including consent withdrawal, please see our Privacy Policy.

Reviewer #1: No

Reviewer #2: No

[NOTE: If reviewer comments were submitted as an attachment file, they will be attached to this email and accessible via the submission site. Please log into your account, locate the manuscript record, and check for the action link "View Attachments". If this link does not appear, there are no attachment files.]

While revising your submission, please upload your figure files to the Preflight Analysis and Conversion Engine (PACE) digital diagnostic tool, https://pacev2.apexcovantage.com/. PACE helps ensure that figures meet PLOS requirements. To use PACE, you must first register as a user. Registration is free. Then, login and navigate to the UPLOAD tab, where you will find detailed instructions on how to use the tool. If you encounter any issues or have any questions when using PACE, please email PLOS at figures@plos.org. Please note that Supporting Information files do not need this step.

# Author comment: Author response to Decision Letter 1

**DOI:** 10.1371/journal.pone.0260271.r004

1 Oct 2021

APPENDIX - Reviewer comments

Reviewer #1

“I would like to see some details in their description chart about how the 3D printing was performed.” 

Response: As suggested by the reviewer, we inserted a column in table 2 (pages 13-15) with information on the 3D printing process. No or limited information was described in the included studies on who prescribed the 3D-printed orthoses and who fabricated the orthoses. However, we were able to obtain information of the way of geometry acquisition, the software used, type of 3D printing, and material used.

Furthermore, on page 11 line 223, we specified that information regarding the 3D printing process is presented in table 2.

---------------------------------------------------------

Reviewer #2

The reviewer indicated that all comments have been thoroughly addressed.

________________________________________

Attachment Submitted filename: Response to reviewers_01.10.21.docx Click here for additional data file.

# Editor report: Decision Letter 2

**DOI:** 10.1371/journal.pone.0260271.r005

**Authors:** David Benjamin Lumenta

8 Nov 2021

Effectiveness of 3D-printed orthoses for traumatic and chronic hand conditions: a scoping review

PONE-D-21-10493R2

Dear Dr. Oud,

We’re pleased to inform you that your manuscript has been judged scientifically suitable for publication and will be formally accepted for publication once it meets all outstanding technical requirements.

Within one week, you’ll receive an e-mail detailing the required amendments. When these have been addressed, you’ll receive a formal acceptance letter and your manuscript will be scheduled for publication.

An invoice for payment will follow shortly after the formal acceptance. To ensure an efficient process, please log into Editorial Manager at http://www.editorialmanager.com/pone/, click the 'Update My Information' link at the top of the page, and double check that your user information is up-to-date. If you have any billing related questions, please contact our Author Billing department directly at authorbilling@plos.org.

If your institution or institutions have a press office, please notify them about your upcoming paper to help maximize its impact. If they’ll be preparing press materials, please inform our press team as soon as possible -- no later than 48 hours after receiving the formal acceptance. Your manuscript will remain under strict press embargo until 2 pm Eastern Time on the date of publication. For more information, please contact onepress@plos.org.

Kind regards,

David Benjamin Lumenta, MD PhD

Academic Editor

PLOS ONE

Additional Editor Comments (optional):

Reviewers' comments:

# Editor report: Acceptance letter

**DOI:** 10.1371/journal.pone.0260271.r006

**Authors:** David Benjamin Lumenta

10 Nov 2021

PONE-D-21-10493R2 

Effectiveness of 3D-printed orthoses for traumatic and chronic hand conditions: a scoping review 

Dear Dr. Oud:

I'm pleased to inform you that your manuscript has been deemed suitable for publication in PLOS ONE. Congratulations! Your manuscript is now with our production department. 

If your institution or institutions have a press office, please let them know about your upcoming paper now to help maximize its impact. If they'll be preparing press materials, please inform our press team within the next 48 hours. Your manuscript will remain under strict press embargo until 2 pm Eastern Time on the date of publication. For more information please contact onepress@plos.org.

If we can help with anything else, please email us at plosone@plos.org. 

Thank you for submitting your work to PLOS ONE and supporting open access. 

Kind regards, 

PLOS ONE Editorial Office Staff

on behalf of

Professor David Benjamin Lumenta 

Academic Editor

PLOS ONE
